# Heterostructures by Templated Synthesis of Layered Double Hydroxide to Modulate the Electronic Structure of Nickel Sites for a Highly Efficient Oxygen Evolution Reaction

**DOI:** 10.1002/smsc.202300294

**Published:** 2024-02-28

**Authors:** Kexin Xu, Haihong Zhong, Xuying Li, Jie Song, Luis Alberto Estudillo‐Wong, Jun Yang, Yongjun Feng, Xiaojuan Zhao, Nicolas Alonso‐Vante

**Affiliations:** ^1^ State Key Laboratory of Chemical Resource Engineering Beijing Engineering Center for Hierarchical Catalysts College of Chemistry Beijing University of Chemical Technology No. 15 Beisanhuan East Road Beijing 100029 China; ^2^ School of Chemistry and Chemical Engineering Hainan University No. 58 Renmin Road Haikou 570228 P. R. China; ^3^ Departamento de Biociencias e Ingeniería CIIEMAD‐IPN Instituto Politécnico Nacional Ciudad de México C.P. 07340 Mexico; ^4^ State Key Laboratory of Multiphase Complex Systems Institute of Process Engineering Chinese Academy of Sciences Beijing 100190 China; ^5^ Institute of High Energy Physics Chinese Academy of Science Beijing 100190 China; ^6^ IC2MP UMR‐CNRS 7285 University of Poitiers Poitiers Cedex F‐86073 France

**Keywords:** electrocatalysts, heterostructure, layered double hydroxides, oxygen evolution reaction, transition metal chalcogenides

## Abstract

The design and development of highly efficient electrocatalysts for oxygen evolution reaction (OER) are critical for renewable energy generation. Ni‐based electrocatalysts are widely used in the water electrolysis process. In this work, heterostructure consisting of selenides and layered double hydroxides (LDH) named (Co, Ni)Se_4_@NiFe‐LDH, are prepared by an LDH‐based strategy, in which the electronic structure of Ni active sites is regulated by interfacial electron interaction. The (Co, Ni)Se_4_@NiFe‐LDH shows an optimized charge distribution of Ni sites and excellent catalytic activity. The effective charge modulation results in lowering the energy barrier of OOH* intermediate formation and adequate adsorption strength of the intermediates on Ni‐active sites, which improves the kinetics of OER. Specifically, the (Co, Ni)Se_4_@NiFe‐LDH only requires an overpotential of 237 mV to reach the current density of 10 mA cm^−2^ under alkaline conditions. The results of this work demonstrate that reasonable engineering of heterostructure is an effective strategy to improve the intrinsic property of OER electrocatalysts for water splitting.

## Graphical abstract

The engineering of double hydroxide heterostructures proves to be an effective strategy to tailor the electronic structure of the active sites, allowing efficient charge modulation towards the reduction of the energy barrier of formation of the OOH* intermediate. Thus, the OER process in alkaline medium, at 10 mA cm_−2_, only requires an overpotential of 237 mV.

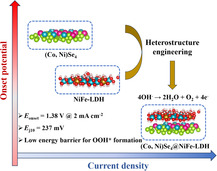

## 1 Introduction

Considering the increasing demand for energy and concern for the environment, high‐efficiency and sustainable energy storage and conversion systems have attracted great interest as they operate using clean and renewable energy sources such as wind energy, solar energy, and hydrogen energy.^[^ ^1^ ^]^ As a promising alternative to non‐renewable fossil fuels, hydrogen has received great attention due to its abundance, high energy density (120–142 MJ kg^−1^), and environmental friendliness. Currently, electrochemical water splitting represents an environmentally friendly method to produce H_2_ from water. However, the anodic reaction, i.e., oxygen evolution reaction (OER), is a slow process involving charge transfer of several electrons/protons and adsorption/desorption steps, which severely hampers the total electricity consumption due to the high overpotential.^[^ ^2^ ^]^ Ru/Ir‐based catalysts have the best catalytic activity for OER.^[^ ^3^ ^]^ However, noble‐metals still have some drawbacks, such as high cost and scarcity. To solve this problem, increasing attention has been paid to explore cost‐effective and abundant alternative materials on earth to satisfy large‐scale hydrogen production by water splitting.

Recently, compounds based on 3*d* transition metals (M = Co, Ni, Fe, Mn, etc.), such as oxides,^[^ ^4^ ^]^ hydroxides,^[^ ^5^ ^]^ nitrides,^[^ ^6^ ^]^ phosphides,^[^ ^7^ ^]^ and chalcogenides^[^ ^8^ ^]^ have been designed and developed. In particular, selenides based on transition metals (M_*x*_Se_*y*_; M = Co, Ni) have attracted great interest due to their relatively high intrinsic activity, low electrical resistivity, and tunable electronic structure.[^7^, ^9^] The high degree of covalency in the M─Se bond helps to facilitate an adequate energy gap with respect to the oxidation and redox potentials of water, in order to accelerate the redox reactions of water at the catalytic sites of M_*x*_Se_*y*_.^[^ ^10^ ^]^ However, although some transition metal selenides have shown efficient catalytic activity for OER, there have been some concerns about their stability under alkaline conditions.^[^ ^11^ ^]^ In contrast, Ni‐based layered double hydroxides (LDHs), can not only catalyze the OER efficiently, but also maintain high structural/chemical‐compositional stability. LDHs with a unique 2D structure, abundant interstratified electrons and channels for intermediate adsorption and desorption show interesting performance in water splitting.^[^ ^12^ ^]^ This type of material possesses especially a large number of edge sites and unsaturated coordination metal sites, which allow their integration with other materials. In addition, the tunable composition (the type of metal cations and the molar ratio of MII/(MII + MIII)) in the host‐sheet of LDHs, and the topological transformation characteristic, enable them to be used as a satisfactory precursor. However, pristine LDHs present a serious problem of re‐stacking, resulting in less exposure of active sites; the low conductivity (10^−13^–10^−17^ S cm^−1^) can also weaken the electron charge transfer process during electrocatalysis. Therefore, heterostructure engineering, i.e., hybridization, is considered an intriguing method to overcome the activity limitations of transition metal selenides and Ni‐based LDHs, as identified to facilitate the performance of global water electrolysis.^[^ ^13^ ^]^ However, the effect of heterostructure on the modulation of electronic structure of Ni sites as catalytic centers for OER remains uncertain. Therefore, it is important to determine the effect of the heterostructure and explain how the Ni sites are electronically modulated to enhance the electrocatalytic activity of OER.

**Figure 1:**
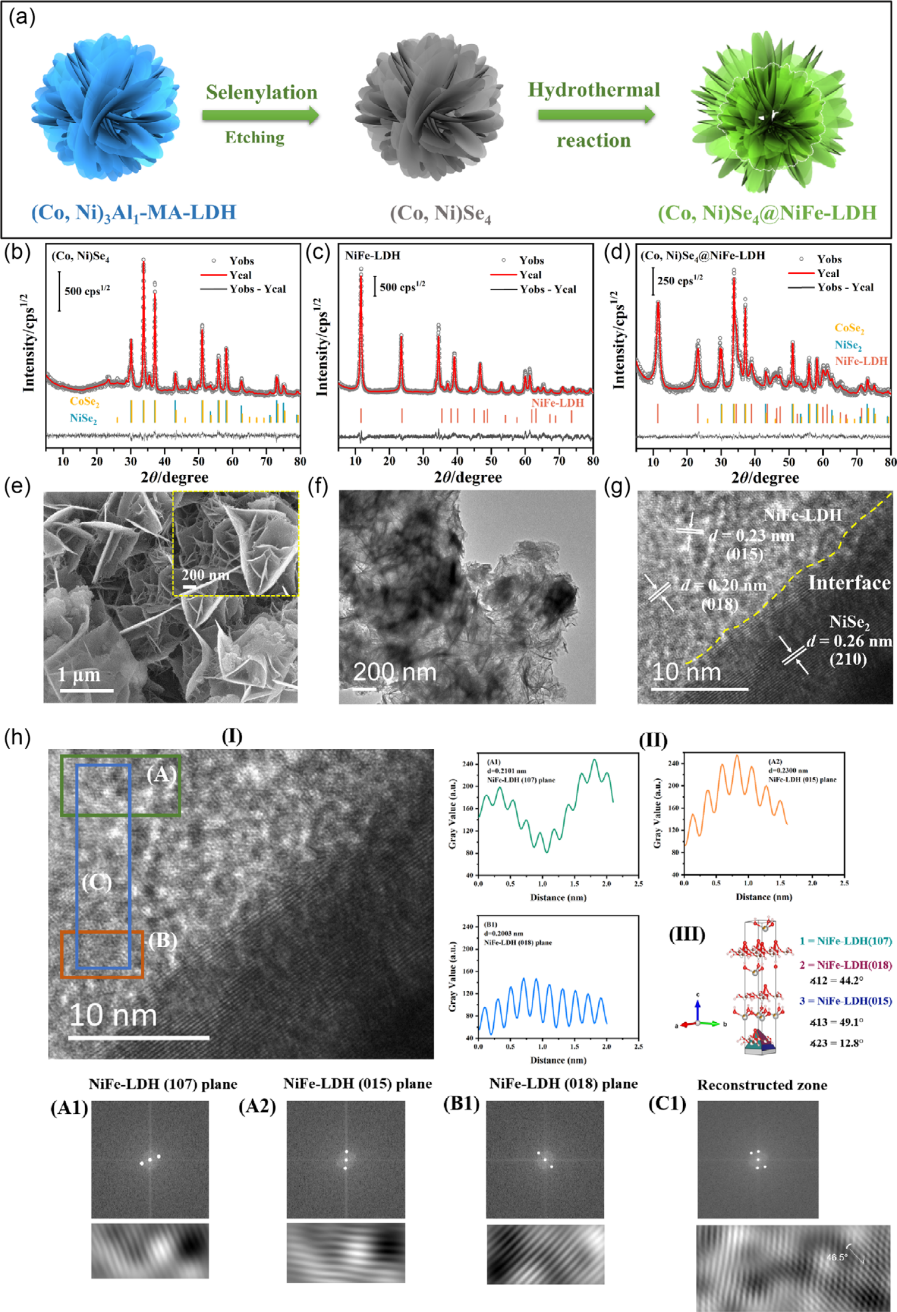
a) schematic illustration of the formation process for the (Co, Ni)Se_4_@NiFe LDH; b–d) power X‐Ray diffraction experimental and fitting patterns for (Co, Ni)Se_4_, NiFe‐LDH, and (Co, Ni)Se_4_@NiFe‐LDH samples in the interval from 3º to 80º/2*θ* and the standard patterns of CoSe_2_, NiSe_2_, NiFe‐LDH. GofF (Rwp%) parameters are 1.12 (7.62), 1.19 (6.86), and 1.47 (9.06) for (Co, Ni)Se_4_, NiFe‐LDH, and (Co, Ni)Se_4_@NiFe‐LDH samples, respectively. Yobs, Ycal and Yobs‐Ycal are the experimental, calculated profile and the difference between them, respectively; e) SEM image and f,g) HRTEM images of the (Co, Ni)Se_4_@NiFe‐LDH; h) HRTEM images of (Co, Ni)Se_4_@NiFe‐LDH (I), the distance planes (II) and the crystal shape for the NiFe‐LDH phase (III). Insets A1, A2, B1 and C1 correspond to the NiFe‐LDH(107), NiFe‐LDH(015), NiFe‐LDH(018) planes, respectively, and the reconstructed zone planes for FFT and inverse FFT, respectively.

In this work, we present an LDH‐templated synthesis strategy to construct selenide/NiFe‐LDH heterostructure as an anodic electrocatalyst for water splitting. In a typical demonstration, (Co, Ni)Se_4_@NiFe‐LDH hydrangea‐like microspheres are prepared by “three‐steps” including etching, selenylation, and hydrothermal treatment, as illustrated in **Figure** **1**a. First, LDH microspheres intercalated with metanilic acid, i.e., (Co, Ni)_3_Al_1_‐MA‐LDH, with hierarchical structure are synthesized by the hydrothermal method as described in our previously work.^[^ ^14^ ^]^ Next, the prepared (Co, Ni)_3_Al_1_‐MA‐LDH microspheres are subjected to heat treatment and alkaline etching to produce the carbonaceous derivate (e‐LDH). Then, (Co, Ni)Se_4_ is obtained by in situ topological transformation of e‐LDH in the presence of a Se source. Finally, NiFe‐LDH nanosheets/nanowires are grown on the surface of (Co, Ni)Se_4_ via hydrothermal reaction to produce (Co, Ni)Se_4_@NiFe‐LDH microspheres. Systematic experiments and density functional theory (DFT) calculations are applied to study the effect of surface/interface electron interaction in a heterostructured electrocatalyst on the modulation of active sites properties. The optimized intrinsic properties, such as enhanced electrical conductivity, modulated electronic structure, and abundant diffusion paths, endows the heterostructured (Co, Ni)Se_4_@NiFe‐LDH catalyst with noteworthy OER performance in alkaline medium compared to that of individual (Co, Ni)Se_4_ and NiFe‐LDH catalysts.

## 2 Results and Discussion

### 2.1 Structure and Morphology Characterizations

The in situ transformation into (Co, Ni)Se_4_ hybrid from (Co, Ni)_3_Al_1_‐MA‐LDH and the formation of (Co, Ni)_3_Al_1_‐MA‐LDH heterostructure are evidenced by the chemical‐compositional, structural, and morphological changes. As shown in Figure S1 (Supporting Information), the diffraction peaks of the (Co, Ni)_3_Al_1_‐MA‐LDH precursor located at 5.76°, 11.46° and 17.08°/2*θ* correspond to the (003), (006), and (009) planes of a typical LDH phase, respectively. The interlayer distance (*d*_003_) is calculated to be 1.53 nm, confirming that the metanilic acid anions are successfully intercalated into the LDH interlamellar.^[^ ^15^ ^]^ Figure 1b–d depict the powder X‐ray diffraction (pXRD) patterns of the (Co, Ni)Se_4_, NiFe‐LDH, and (Co, Ni)Se_4_@NiFe‐LDH samples in the 3–80°/2*θ* interval, respectively. The XRD pattern of (Co, Ni)Se_4_ sample shows a series of diffraction peaks, which can be assigned to CoSe_2_ (JCPDS No. 09‐0234) and NiSe_2_ (JCPDS No. 41‐1495), indicating that the Co, Ni‐based composite derived from the precursor (Co, Ni)_3_Al_1_‐MA‐LDH has been successfully converted to metal selenides after selenylation treatment. In particular, both NiSe_2_ and CoSe_2_ phases occur in cubic crystalline systems with Pa‐3 space group. In the XRD pattern of NiFe‐LDH, several main peaks locate around 11.41°, 23.01°, 34.05°, 38.89°, 46.32°/2*θ* are attributed to the lattice planes (003), (006), (012), (015), (018) of rhombohedral LDH phase with trigonal crystalline system and R‐3m space group (JCPDS No. 40‐0215), respectively. Along with NiSe_2_ and CoSe_2_, NiFe‐LDH phase is also observed in the (Co, Ni)Se_4_@NiFe‐LDH sample, suggesting the formation of selenides and LDH composites.

To obtain further structural information, the Rietveld refinement method (RRM) was applied to all samples. In this case, the standard reference data of COD#901‐2537 and COD#901‐2536 from the open crystallography database (COD) for NiSe_2_ and CoSe_2_, respectively, were used. The microstructural properties of the samples are summarized in **Table** **1**. RRM analysis again verifies the existence of *c*‐CoSe_2_ and *c*‐NiSe_2_ phases in the (Co, Ni)Se_4_ sample. In the case of (Co, Ni)Se_4_@NiFe‐LDH sample, the XRD pattern is associated with cubic CoSe_2_, cubic NiSe_2_, and rhombohedral NiFe‐LDH phases. It is noteworthy that no change in phase composition or crystalline system occurs during the formation of the (Co, Ni)Se_4_@NiFe‐LDH composite. However, when NiFe‐LDH is introduced, the lattice parameters of CoSe_2_ and NiSe_2_ phases decrease from 5.9072 to 5.8880 Å and 5.9321 to 5.9207 Å, respectively. As for the NiFe‐LDH phase in the (Co, Ni)Se_4_@NiFe‐LDH, an expansion in the [001] direction is observed (i.e., the parameter c increases from 22.6689 to 22.9877 Å). Interestingly, a decreasing trend in the average crystal size of all phases, including CoSe_2_, NiSe_2_, and NiFe‐LDH phases, is observed after the formation of the (Co, Ni)Se_4_@NiFe‐LDH composite material. These phenomena can be attributed to the fact that the NiFe‐LDH phase has grown in situ on the surface of (Co, Ni)Se_4_ by hydrothermal reaction, resulting in an intimate contact between NiFe‐LDH and (Co, Ni)Se_4_ in the (Co, Ni)Se_4_@NiFe‐LDH sample. As a result, the average crystallite size is reduced from 29.1 ± 0.18 to 10.8 ± 1.1 nm, in the NiFe‐LDH phase. In addition, the amorphous NiFe‐LDH phase is formed at the interface of (Co, Ni)Se_4_ and NiFe‐LDH due to the expansion of parameter *c*. This observation is also confirmed by a broader diffraction peak in the (003) plane of NiFe‐LDH in the (Co, Ni)Se_4_@NiFe‐LDH sample compared to that of NiFe‐LDH.

**Table 1:** Microstructural properties obtained by RRM for (Co, Ni)Se_4_, NiFe‐LDH, and (Co, Ni)Se_4_@NiFe‐LDH

Samples	Phases	Lattice parameter [Å]	<*d*> [nm]
(Co, Ni)Se_4_	NiSe_2_ (Pa‐3) CoSe_2_ (Pa‐3)	*a* = 5.9321 (5E‐4) *a* = 5.9092 (1E‐3)	87.8(2.9) 43.3(1.5)
NiFe‐LDH	NiFe‐LDH (R‐3m)	*a* = *b* = 3.0826 (2E‐4) *c* = 22.6689 (3E‐3)	29.1(0.18)
(Co, Ni)Se_4_@NiFe‐LDH	NiSe_2_ (Pa‐3) CoSe_2_ (Pa‐3) NiFe‐LDH (R‐3m)	*a* = 5.9263 (5E‐4) *a* = 5.8880 (5E‐3) *a* = *b* = 3.0862 (6E‐4) *c* = 22.9877 (6E‐3)	64.4(2.6) 10.7(0.6) 10.8(1.1)

The morphologies of the LDH‐based precursors, (Co, Ni)Se_4_, NiFe‐LDH, and (Co, Ni)Se_4_@NiFe‐LDH catalysts were investigated by scanning electron microscope (SEM) and transmission electron microscope (TEM), cf. Figure 1e,f, and S2, Supporting Information. Clearly, the (Co, Ni)_3_Al_1_‐MA‐LDH precursor exhibits a hydrangea‐like morphology with an average diameter of 2 μm, and is composed of numerous interconnected LDHs nanosheets. After carbonization and alkaline‐etching treatment, the hydrangea‐like structure is maintained in the e‐LDH sample, as shown in Figure S2 (Supporting Information). It can be seen that the (Co, Ni)Se_4_ sample, originated from e‐LDH after chemical conversion with Se powder at a relatively high calcination temperature, still maintains the original hydrangea‐like morphology (Figure S2c, Supporting Information), but a small number of agglomerated nanoparticles with an average size of 50–100 nm appear on the surface of nanosheets. As shown in Figure 1e, the (Co, Ni)Se_4_@NiFe‐LDH sample exhibits a heterostructure with NiFe‐LDH nanosheets/nanowires growing around the (Co, Ni)Se_4_ surface. The ultrathin nanosheet structure allows more active sites to be exposed. The high‐resolution TEM image of the (Co, Ni)Se_4_@NiFe‐LDH shows an interface between (Co, Ni)Se_4_ and the NiFe‐LDH, cf. Figure 1g. The lattice spacing of 0.26 nm may correspond to the (210) plane of NiSe_2_, while the lattice spacings of 0.20 and 0.23 nm correspond to the (018) plane and (015) plane of NiFe‐LDH, respectively. More specifically, the electron diffraction patterns obtained by fast Fourier transform (FFT) in (A), (B), and (C) zones, are shown in Figure 1h. First, zone (A) is composed of two planes, in which NiFe‐LDH (015) and NiFe‐LDH (107) planes coexist. Lattice spacings of 0.21 and 0.23 nm correspond to the (107) plane and (015) plane of NiFe‐LDH, respectively, confirming the inverse FFT (Figure 1h–II). The zone (B) depicts the NiFe‐LDH (018) plane with the lattice distance of 0.20 nm. All these values are verified by the simulated crystal unit cell, cf. Figure 1h–III. Using Vesta software, the unit cell and crystal shape were simulated using the microstructural values obtained by RRM, cf. Figure 1h–III. The results show that the angle between NiFe‐LDH (107) and NiFe‐LDH (018) or NiFe‐LDH (015) planes are 44.2° and 49.1°, respectively. The angle between NiFe‐LDH (015) and NiFe‐LDH (018) is 12.8°, difficult to measure because they represent a family of planes, i.e., the (01l) planes. However, the angle, obtained in the reconstructed zone (zone C, Figure 1h–C1), presents a value of 46.5°, which is between the values 44.2° and 49.1°. These values confirmed that those planes coexist in the sample. Besides, energy dispersive X‐ray spectroscopy (EDX) mapping images evidence the uniform distribution of Co, Se, Ni, and Fe elements throughout the heterostructure, especially Co and Se elements are mainly dispersed in the core of the (Co, Ni)Se_4_@NiFe‐LDH microsphere (Figure S3, Supporting Information).

**Figure 2:**
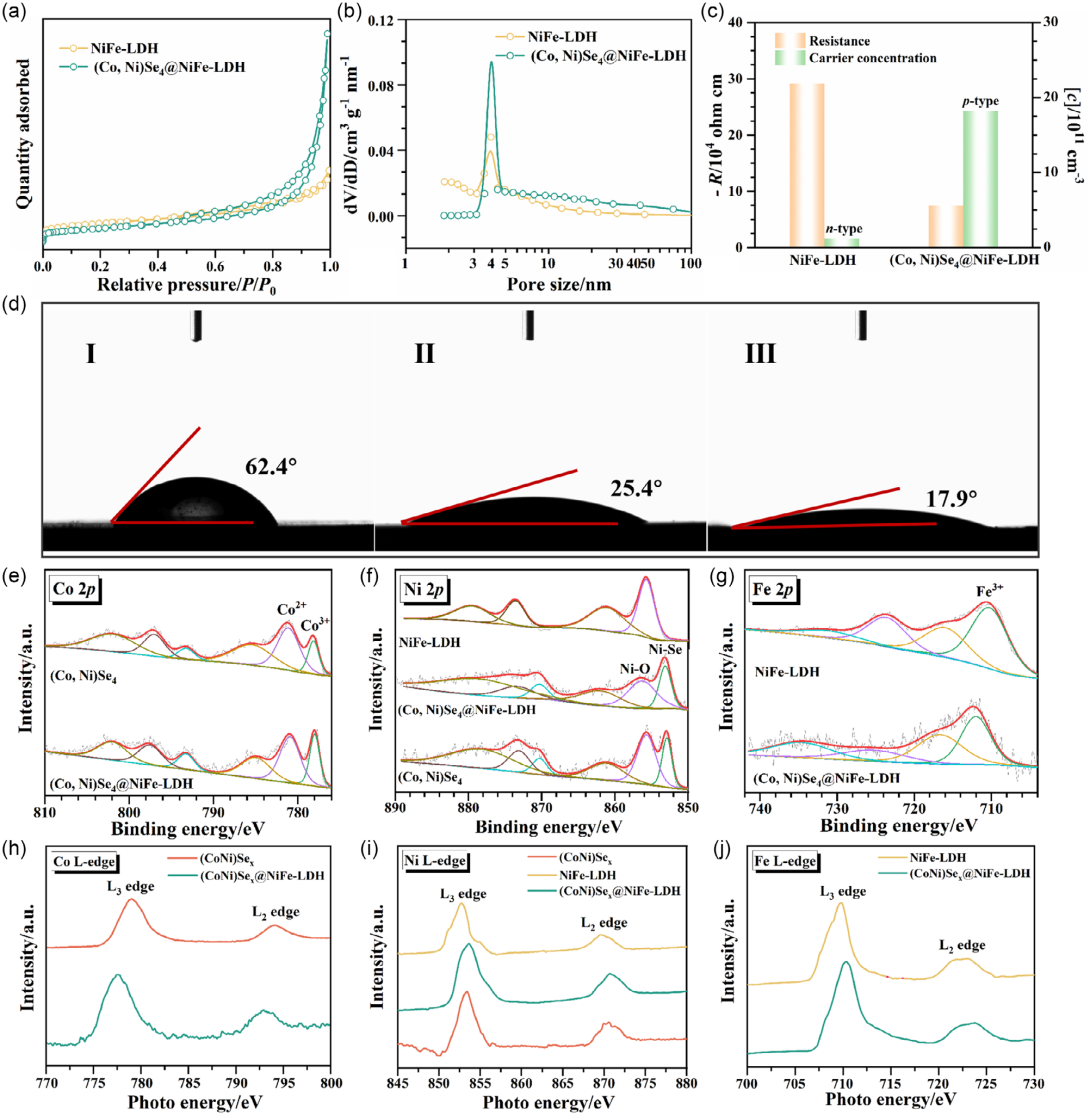
a) N_2_ adsorption‐desorption isotherms; b) pore‐size distribution curves; c) Hall coefficient and corresponding carrier concentration of (Co, Ni)Se_4_, NiFe‐LDH, and (Co, Ni)Se_4_@NiFe‐LDH catalysts; d) Contact angle measurements for (I) (Co, Ni)Se_4_, (II) NiFe‐LDH, and (III) (Co, Ni)Se_4_@NiFe‐LDH; XPS spectra of e) Co 2*p*; f) Ni *2p*; g) Fe 2*p*; *s*‐XAS spectra of h) Co L‐edge; i) Ni L‐edge; j) Fe L‐edge in (Co, Ni)Se_4_, NiFe‐LDH, and (Co, Ni)Se_4_@NiFe‐LDH samples.

The FTIR spectra of the (Co, Ni)Se_4_, NiFe‐LDH, and (Co, Ni)Se_4_@NiFe‐LDH samples are shown in Figure S4 (Supporting Information). All these samples show broad vibrational absorption peaks at 3446 cm^−1^, corresponding to the O─H stretching vibration. The peak at 1622 cm^−1^ can be assigned to the H─OH vibration of water molecules adsorbed on the surface of the samples.^[^ ^16^ ^]^ The FTIR spectra of NiFe‐LDH and (Co, Ni)Se_4_@NiFe‐LDH show characteristic adsorption peaks at ≈1362 cm^−1^, which are attributed to carbonate ions in the interlayer region of LDH, demonstrating the existence of NiFe‐LDH on the surface of the (Co, Ni)Se_4_@NiFe‐LDH heterostructure. The peaks located around 601cm^−1^ and 470 cm^−1^ are attributed to the stretching vibration of Ni–OH and metal‐Se coordination bond, respectively.^[^ ^17^ ^]^ **Figure** **2**a,b show typical N_2_ adsorption‐desorption isotherms and pore‐size distribution curves for NiFe‐LDH and (Co, Ni)Se_4_@NiFe‐LDH, respectively. Both isotherms show typical H3‐type hysteresis loops, indicating the presence of mesopores.^[^ ^18^ ^]^ The (Co, Ni)Se_4_@NiFe‐LDH sample has a higher specific surface area of 155.25 m^2^ g^−1^ than that of NiFe‐LDH (118.77 m^2^ g^−1^), suggesting that the former may provide more active sites during electrocatalysis.

To investigate the electrical conductivity and charge transport property of the NiFe‐LDH and (Co, Ni)Se_4_@NiFe‐LDH samples, the Hall coefficient (*R*_H_) resistance was measured, as illustrated in Figure 2c. It is noteworthy that the electrical resistivity of 7.47 × 10^4^ Ω cm^−1^ of (Co, Ni)Se_4_@NiFe‐LDH is approximately one magnitude lower than that of NiFe‐LDH (2.91 × 10^5^ Ω cm^−1^), suggesting an enhancement of the conductivity with the formation of the heterostructure. NiFe‐LDH and (Co, Ni)Se_4_@NiFe‐LDH show negative and positive *R*_H_ at 300 K, respectively, indicating that the former is an n‐type semiconductor and the latter is a p‐type semiconductor. Their majority charge carriers are electrons and holes, respectively. This result demonstrates that there exists a heterojunction interface between NiFe‐LDH and (Co, Ni)Se_4_ in the (Co, Ni)Se_4_@NiFe‐LDH sample. As expected, the carrier concentration of the (Co, Ni)Se_4_@NiFe‐LDH increases 15 times compared to that of NiFe‐LDH, which means a significant enhancement of the conductivity of (Co, Ni)Se_4_@NiFe‐LDH heterostructure. In addition, the wettability of the electrode is also an important factor driving the OER process at high current density, since continuous and violent bubbles will be generated during the OER process at high current density, which could adhere to the catalyst surface and thus block the active sites by hindering the charge and mass transfer at the solid–liquid interface. As shown in Figure 2d, the contact angles of (Co, Ni)Se_4_, NiFe‐LDH and (Co, Ni)Se_4_@NiFe‐LDH samples are 62.4°, 25.4°, and 17.9°, respectively. This means that the (Co, Ni)Se_4_@NiFe‐LDH exhibits more obvious hydrophilicity compared to the other two samples, which favors the wetting of the electrolyte and the outflow of oxygen bubbles, thus improving the activity and stability of the OER especially at high current density.^[^ ^19^ ^]^

### 2.2 Chemical State and Structure Analysis

X‐ray photoelectron spectroscopy (XPS) was performed to study the chemical state of (Co, Ni)Se_4_, NiFe‐LDH and (Co, Ni)Se_4_@NiFe‐LDH samples. The Co XPS spectrum of (Co, Ni)Se_4_, in Figure 2e, shows spin‐orbit coupling at ≈781.2 and ≈797.1 eV, corresponding to Co 2*p*_3/2_ and Co 2*p*_1/2_, respectively, suggesting that the valence of Co is 2+.^[^ ^20^ ^]^ The peaks located at 778.1 ± 0.2 eV indicate the existence of Co^3+^. Compared with those of (Co, Ni)Se_4_, the binding energies of the XPS peaks in the (Co, Ni)Se_4_@NiFe‐LDH heterostructure show a negative shift of ≈0.2 eV, indicating a higher electron density of the Co element in the heterostructure. As for the Ni 2*p* spectra, a pair of peaks at ≈853.1 and ≈870.3 eV accompanied by two satellite peaks located at ≈862.4 and ≈880.1 eV, can be assigned to Ni 2*p*_3/2_ and Ni 2*p*_1/2_, which belong to the Ni─O bond, Figure 2f. The other two peaks centered at ≈853.1 and ≈871.3 eV are attributed to Ni─Se bonding.^[^ ^21^ ^]^ It is evident that the binding energies of Ni─Se and Ni─O peaks are higher in the (Co, Ni)Se_4_@NiFe‐LDH heterostructure than those of (Co, Ni)Se_4_ and NiFe‐LDH. The above analysis implies that there is an electron‐transfer interaction between Co and Ni elements after the formation of the heterostructure. The XPS spectra of Fe 2*p* in NiFe‐LDH and (Co, Ni)Se_4_@NiFe‐LDH certify a pair of spin‐orbit couplings, i.e., Fe 2*p*_3/2_ and Fe 2*p*_1/2_, and their satellite peaks, cf. Figure 2g. The XPS signals centered at ≈710.5 eV, are attributed to the Fe^3+^ feature in the Fe 2*p*_3/2_ peak.^[^ ^22^ ^]^ In contrast to NiFe‐LDH, the Fe^3+^ peak of the (Co, Ni)Se_4_@NiFe‐LDH is shifted to a higher binding energy, implying the decrease of the electron density of Fe element. As is evident from previous theoretical and experimental results, high‐valence Ni and Fe would reduce energy barriers and achieve high catalytic activity for oxygen evolution.^[^ ^14^, ^23^ ^]^ In Figure S5 (Supporting Information), after deconvolution of the Se 3*d* spectra, the XPS signals centered at ≈54.4, and ≈55.3 eV, are attributed to Se 3*d*_5/2_ and Se 3*d*_3/2_, respectively, while the peak at 59.3 eV can be assigned to the Se─O bond due to the surface oxidation of Se species exposed to air.^[^ ^24^ ^]^ Similarly, it can be seen that there is a shift in the binding energy of the Se XPS peaks, which is evidence of a change in the electronic structure of the Se element in the heterostructure.

Due to increased sensitivity to elemental composition, chemical state and orbital, synchrotron radiation soft X‐ray absorption spectroscopy (*s*‐XAS) can directly measure the 3*d* states of transition metals, making it a powerful tool for investigating occupied and unoccupied states near the Fermi level.^[^ ^25^ ^]^ In the *s*‐XAS spectrum of the Co L‐edge of (Co, Ni)Se_4_, there are two strong peaks around 779 and 794 eV assigned to L_3_ and L_2_‐edge originating from the electronic transitions of Co 2*p* core electrons, which are split by the spin orbit interaction of the hybridized Co 2*p* core level with the Se 3*p* orbital level, Figure 2h.^[^ ^26^ ^]^ Moreover, it is noteworthy that the L_3_ and L_2_‐edge peaks of (Co, Ni)Se_4_@NiFe‐LDH are shifted to lower energies compared with those of (Co, Ni)Se_4_, showing that there are fewer unoccupied 3*d* states in the former, which could be attributed to the strong interaction between (Co, Ni)Se_4_ and NiFe‐LDH. In Figure 2i, compared to (Co, Ni)Se_4_ and NiFe‐LDH, the L_3_ and L_2_‐edge peaks of Ni of (Co, Ni)Se_4_@NiFe‐LDH show a positive shift toward higher photo energies, revealing the increase in the oxidation valence‐state of the Ni element. Similar phenomena occur in the case of the *s*‐XAS spectrum of the L‐edge of Fe, as shown in Figure 2j. The above *s*‐XAS results agree with those of XPS, finding that the heterointerface can effectively regulate the electronic structure of transition metals.

**Figure 3:**
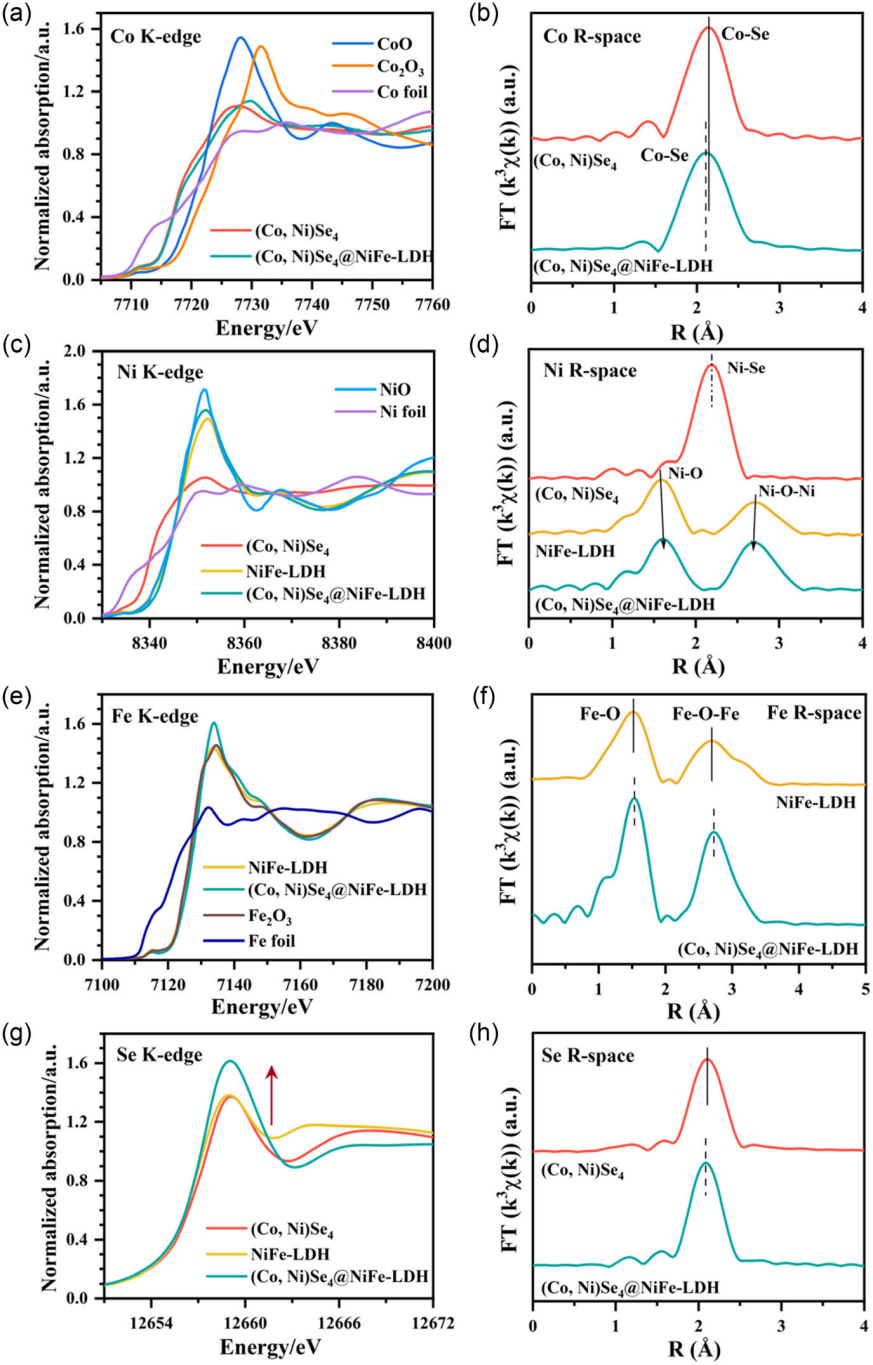
XANES spectra of the normalized a) Co *K*‐edge; b) Ni *K*‐edge; c) Fe *K*‐edge; d) Se *K*‐edge spectra of (Co, Ni)Se_4_, NiFe‐LDH, and (Co, Ni)Se_4_@NiFe‐LDH catalysts and the standards; EXAFS spectra at the e) Co *K*‐edge; f) Ni *K*‐edge; g) Fe *K*‐edge and h) Se *K‐*edge.

In addition, X‐ray absorption near‐edge structure (XANES) and Fourier transform extended X‐ray absorption fine structure (FT‐EXAFS) analyses were carried out to obtain more details on the chemical valence state and coordination environment of the elements Co, Ni, Fe, and Se in the (Co, Ni)Se_4_@NiFe‐LDH heterostructure, cf. **Figure** **3**. All fitting results are summarized in Table S1–S4, Supporting Information. The K‐edge XANES spectra of Co, in Figure 3a, show that the Co pre‐edge positions of (Co, Ni)Se_4_ and (Co, Ni)Se_4_@NiFe‐LDH lie between those of CoO and Co foil, although relative closer to CoO. This indicates the existence of positively charged Co atoms (0 < *δ* < 2). FT‐EXAFS spectra show prominent peaks at 2.11 ± 4 Å, which are typical of Co‐Se scattering, Figure 3b. Compared with (Co, Ni)Se_4_, the Co‐Se peak for (Co, Ni)Se_4_@NiFe‐LDH shifts to a smaller radial distance, demonstrating the contraction of the Co─Se bond and the interfacial effect between (Co, Ni)Se_4_ and NiFe‐LDH in the heterostructure on the local atomic arrangement of the Co site. Similarly, the average chemical valence state of Ni atoms in the (Co, Ni)Se_4_@NiFe‐LDH is +2, as shown in Figure 3c.^[^ ^27^ ^]^ In the K‐edge EXAFS of Ni in R space, Figure 3d, the main coordination peak of (Co, Ni)Se_4_ at 2.19 Å corresponds to the Ni─Se bond coordination, while the two dominant peaks located at 1.58 and 2.71 Å in NiFe‐LDH correspond the first and the second coordination shells of Ni─O and Ni─O─Ni bonds, respectively.^[^ ^28^ ^]^ However, no distinct Ni–Se peak is observed in (Co, Ni)Se_4_@NiFe‐LDH. Compared to the Ni─O and Ni─O─Ni bonds of NiFe‐LDH, a strained lattice with a larger Ni─O bond and a shorter Ni─O─Ni bond is observed in (Co, Ni)Se_4_@NiFe‐LDH. Figure 3e shows the K‐edge XANES spectra of Fe, which reveals an average valence of +3 (Fe^3+^) in both NiFe‐LDH and (Co, Ni)Se_4_@NiFe‐LDH. The corresponding FT‐EXAFS spectra show three prominent peaks at ≈1.54, ≈2.72 and ≈3.13 Å, which are Fe─O, Fe─O─Fe, and Fe─O─Fe coordination paths, respectively, Figure 3f. The K‐edge absorption of Se in (Co, Ni)Se_4_@NiFe‐LDH is slightly shifted to lower energy relative to other two counterparts, agreeing with the aforementioned XPS results and indicating a higher negative charge of Se in (Co, Ni)Se_4_@NiFe‐LDH.

### 2.3 OER Electrocatalytic Performance Evaluation

**Figure 4:**
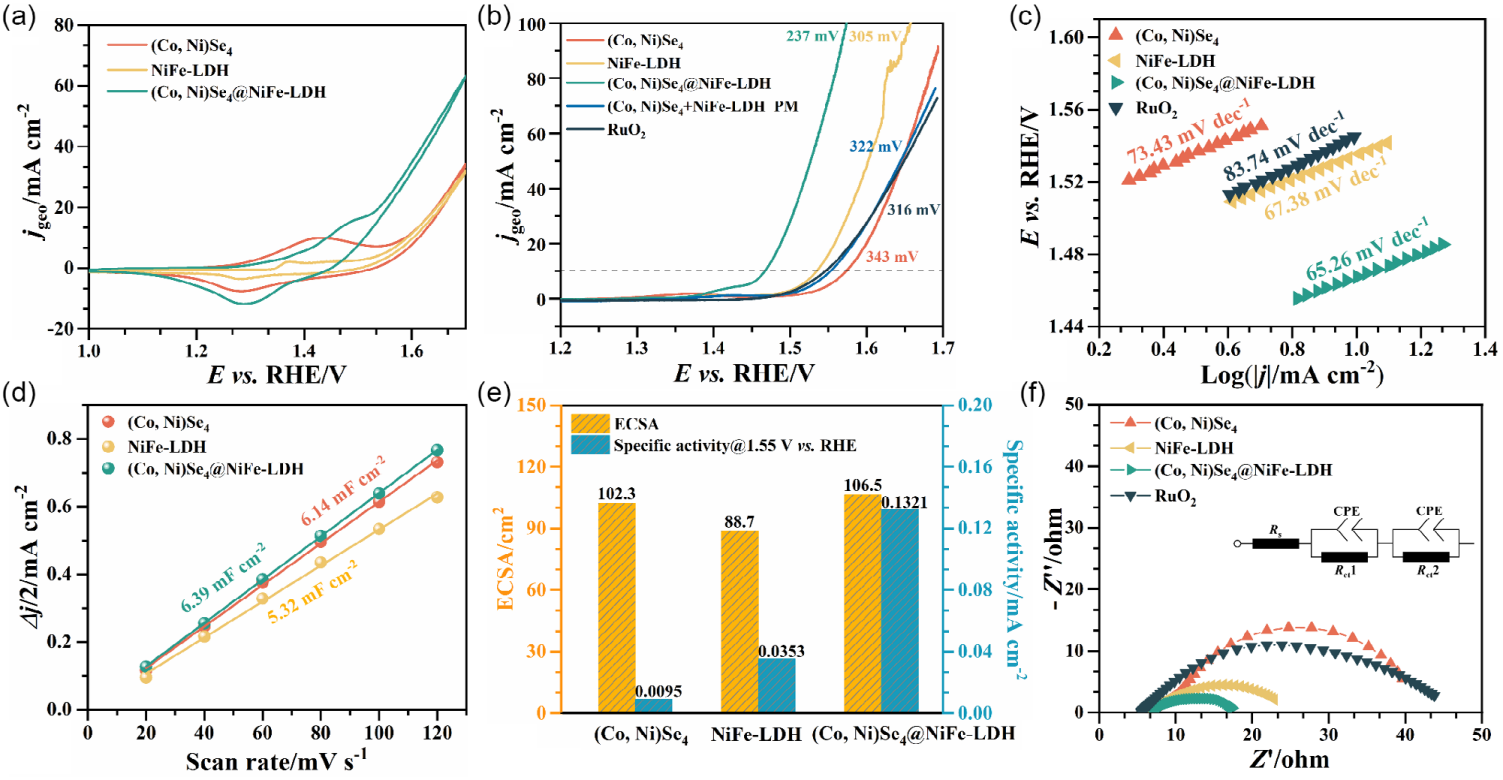
Oxygen evolution electrocatalysis. a) CV curves; b) LSV polarization curves; c) corresponding Tafel plots; d) double‐layer capacitance (*C*_dl_) plots; e) calculated ECSA and specific activity of the catalysts in 1 m KOH solution; f) Nyquist plots with a fitted equivalent circuit.

The OER activities of (Co, Ni)Se_4_, NiFe‐LDH, and (Co, Ni)Se_4_@NiFe‐LDH electrocatalysts were evaluated by linear sweep voltammetry (LSV) and cyclic voltammetry (CV) using RDE in 1 M KOH electrolyte, and 85% *iR*‐corrected was used for LSV measurements. Commercial RuO_2_ was also tested under the same condition as a reference OER catalyst. **Figure** **4**a shows the CV curves of (Co, Ni)Se_4_, NiFe‐LDH, and (Co, Ni)Se_4_@NiFe‐LDH electrocatalysts with a couple of redox peaks, respectively. In the case of the (Co, Ni)Se_4_@NiFe‐LDH electrode, the redox charge under a set of distinct redox peaks assigned to the Co^2+^/Co^3+^ and/or Ni^2+^/(Ni^3+^)Ni^4+^, becomes slightly larger, which can be explained by more exposed active sites.^[^ ^20^, ^29^ ^]^ The electrocatalyst (Co, Ni)Se_4_@NiFe‐LDH shows the best OER catalytic activity with the lowest onset potential of 1.38 V versus RHE@2 mA cm^−2^ and the lowest overpotential of 237 mV to reach a current density of 10 mA cm^−2^ (*η*_10_ = 237 mV), cf. Figure 4b. The trend of OER activity in these electrocatalysts follows the order: (Co, Ni)Se_4_@NiFe‐LDH (*η*_10_ = 237 mV) >NiFe‐DH (*η*_10_ = 305 mV) > (Co, Ni)Se_4_ (*η*_10_ = 343 mV) > RuO_2_ (*η*_10_ = 316 mV), as summarized in Figure S6, Supporting Information. In addition, (Co, Ni)Se_4_@NiFe‐LDH electrocatalyst (*m*_selenide_ = 0.10 g, *M*_Ni_/*M*_Fe_ = 3:1) displays the best OER activity among these catalysts with different selenide/LDH or Ni/Fe ratio (Figure S7, Supporting Information). Furthermore, to elucidate the effect of interfacial interaction on the OER, the (CoNi)Se_4_ + NiFe‐LDH physical mixture (PM) was prepared by simply mechanical mixing, which shows a higher *η*_10_ of 322 mV compared to that of the (Co, Ni)Se_4_@NiFe‐LDH heterostructure, demonstrating the important role of chemical bonding at the interface. The OER activity of the (Co, Ni)Se_4_@NiFe‐LDH electrocatalyst is good among the most non‐noble‐metal‐based heterostructure catalysts reported in the literature, Figure S8, Table S5, Supporting Information.

The Tafel slope was obtained from the LSV polarization curve to gain insight into the OER kinetics, cf. Figure 4c. The Tafel slope of (Co, Ni)Se_4_@NiFe‐LDH (65.26 mV dec^−1^) is lower than that of (Co, Ni)Se_4_ (73.43 mV dec^−1^), NiFe‐LDH (67.38 mV dec^−1^) and RuO_2_ (83.74 mV dec^−1^). The small Tafel slope of (Co, Ni)Se_4_@NiFe‐LDH indicates fast OER kinetics, certainly attributed to the increase in the electrochemical active surface area. Furthermore, a Tafel slope close to 40 mV dec^−1^, suggests that the formation of OOH* or OO* intermediates is most likely the rate‐determining step.^[^ ^30^ ^]^ To evaluate the electrochemical active surface area (ECSA) of the electrocatalysts, capacitive currents were recorded from CV curves in a narrow potential window (where no Faradaic processes were observed) at various scan rates, Figure S9, Supporting Information. The ECSA, associated with the number of active sites, is determined by measurements of the double‐layer capacitance (*C*_dl_). The ECSA value is calculated based on the equation: ECSA =*C*_dl_/*C*_s_, where *C*_s_ is 40 μF cm^−2^. As shown in Figure 4d, the *C*_dl_ of (Co, Ni)Se_4_@NiFe‐LDH, (Co, Ni)Se_4_ and NiFe‐LDH is calculated to be 6.39, 6.14, 5.32 mF cm^−2^, respectively. The ECSA of (Co, Ni)Se_4_@NiFe‐LDH is 106.5 cm^2^, higher than those of (Co, Ni)Se_4_ (102.3 cm^2^), and NiFe‐LDH (88.7 cm^2^), indicating higher exposure of actives sites on (Co, Ni)Se_4_@NiFe‐LDH and (Co, Ni)Se_4_. In addition, the specific activity is obtained by normalizing the current with the ECSA. Significantly, as shown in Figure S10, Supporting Information, (Co, Ni)Se_4_@NiFe‐LDH still shows much lower overpotential than those of (Co, Ni)Se_4_ and NiFe‐LDH. At 1.55 V, the specific activity of (Co, Ni)Se_4_@NiFe‐LDH is almost 4 and 14 times higher than those of (Co, Ni)Se_4_ and NiFe‐LDH, respectively, illustrating the higher intrinsic OER activity of (Co, Ni)Se_4_@NiFe‐LDH due to the heterostructure. The fast kinetics of the (Co, Ni)Se_4_@NiFe‐LDH is also verified by electrochemical impedance spectroscopy (EIS). Figure 4f depicts the Nyquist plots and the equivalent circuit model. The (Co, Ni)Se_4_@NiFe‐LDH provides a charge transfer resistance (*R*_ct_) of 17.72 Ω, much lower than those of (Co, Ni)Se_4_ (41.54 Ω), NiFe‐LDH (24.85 Ω), and RuO_2_ (45.74 Ω), indicating a higher electron transport capacity and conductivity of the heterostructure.

**Figure 5:**
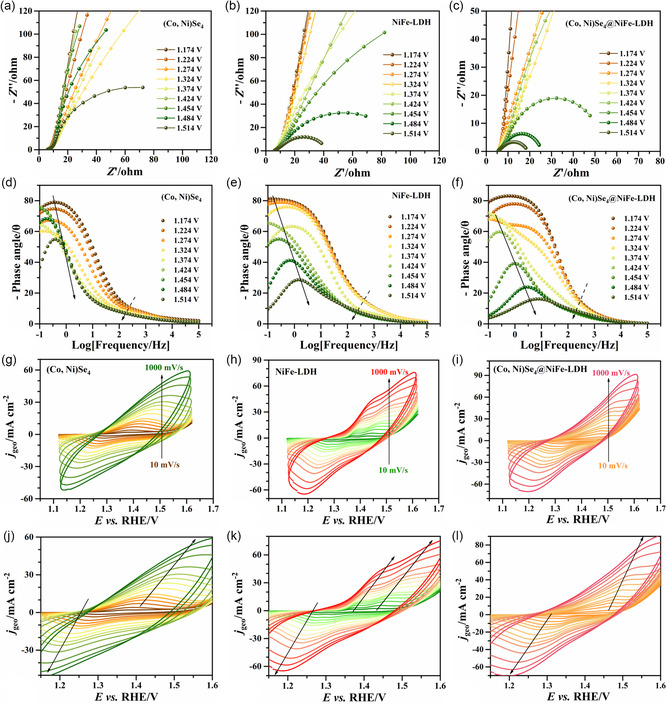
a–c) Nyquist plots and d–f) Bode phase plots of (Co, Ni)Se_4_, NiFe‐LDH, and (Co, Ni)Se_4_@NiFe‐LDH catalysts in 1 m KOH solution under different potentials in the interval from 1.174 to 1.514 V versus RHE, respectively; g–i) CV curves and j–l) magnified CV curves of (Co, Ni)Se_4_, NiFe‐LDH, and (Co, Ni)Se_4_@NiFe‐LDH catalysts at different scan rates (ν): 10, 20, 40, 60, 80, 100, 150, 200, 250, 300, 400, 600, 800, and 1000 mV s^−1^ (from the inner to outer cycle), respectively.

In order to investigate the charge‐transfer resistance and catalytic kinetics of all the samples during the OER process, EIS measurements are performed at different applied electrode potentials, cf. **Figure** **5**a–c. As for the electrocatalyst (Co, Ni)Se_4_@NiFe‐LDH, the Nyquist plots show almost vertical lines at the low potentials from 1.174 to 1.424 V versus RHE, suggesting infinite *R*_ct_. When *E* > 1.424 V, semicircles appear, indicating that oxygen electrocatalysis takes place on (Co, Ni)Se_4_@NiFe‐LDH catalyst. The initial OER potential of (Co, Ni)Se_4_@NiFe‐LDH is 1.454 V, while those of (Co, Ni)Se_4_ and NiFe‐LDH are 1.484 and 1.514 V, respectively, which indicates that the former has higher OER kinetics. Furthermore, the Bode plots of all catalysts show that the phase angle (*θ*) changes with frequency, cf. Figure 5d–f. In line with the Nyquist data, with the anodic applied electrode potential one observes, in the frequency range from 0.1 to 1000 Hz, that the decrease in phase and decrease in the time constant represents the capacitive (high resistance) to dissipative (low resistance) transition, thus relevant for charge transport at the interface (Figure S11, Supporting Information).^[^ ^31^ ^]^ Figure 5g–i show the CV curves of three samples at scan rates range from 10 to 1000 mV s^−1^. Two anodic peaks are found in NiFe‐LDH along with the increased scan rate from the magnified CV curves of NiFe‐LDH, Figure 5k, which can be attributed to the oxidation processes: Ni^2+^ → (Ni^3+^)Ni^4+^ and/or Fe^3+^ → Fe^4+^. However, in Figure 5L, the (Co, Ni)Se_4_@NiFe‐LDH presents only one set of distinct pre‐OER redox feature, but a much higher current density compared to those of NiFe‐LDH, demonstrating a higher electrochemical redox capability of (Co, Ni)Se_4_@NiFe‐LDH due to the modified electronic structure.

In addition, in situ Raman technique was employed to reveal the evolution of the composition of (Co, Ni)Se_4_@NiFe‐LDH during the OER process. As the applied electrode potential increases from 1.20 to 1.70 V versus RHE, the intensity of the (Co, Ni)Se_4_ and NiFe‐LDH bands in the (Co, Ni)Se_4_@NiFe‐LDH gradually decreases. At 1.40 V versus RHE, a Raman band of NiOO^−^ (superoxide species) appears, cf. Figure S12, Supporting Information, which is associated with the partial oxidation of NiFe‐LDH to form NiOOH.

**Figure 6:**
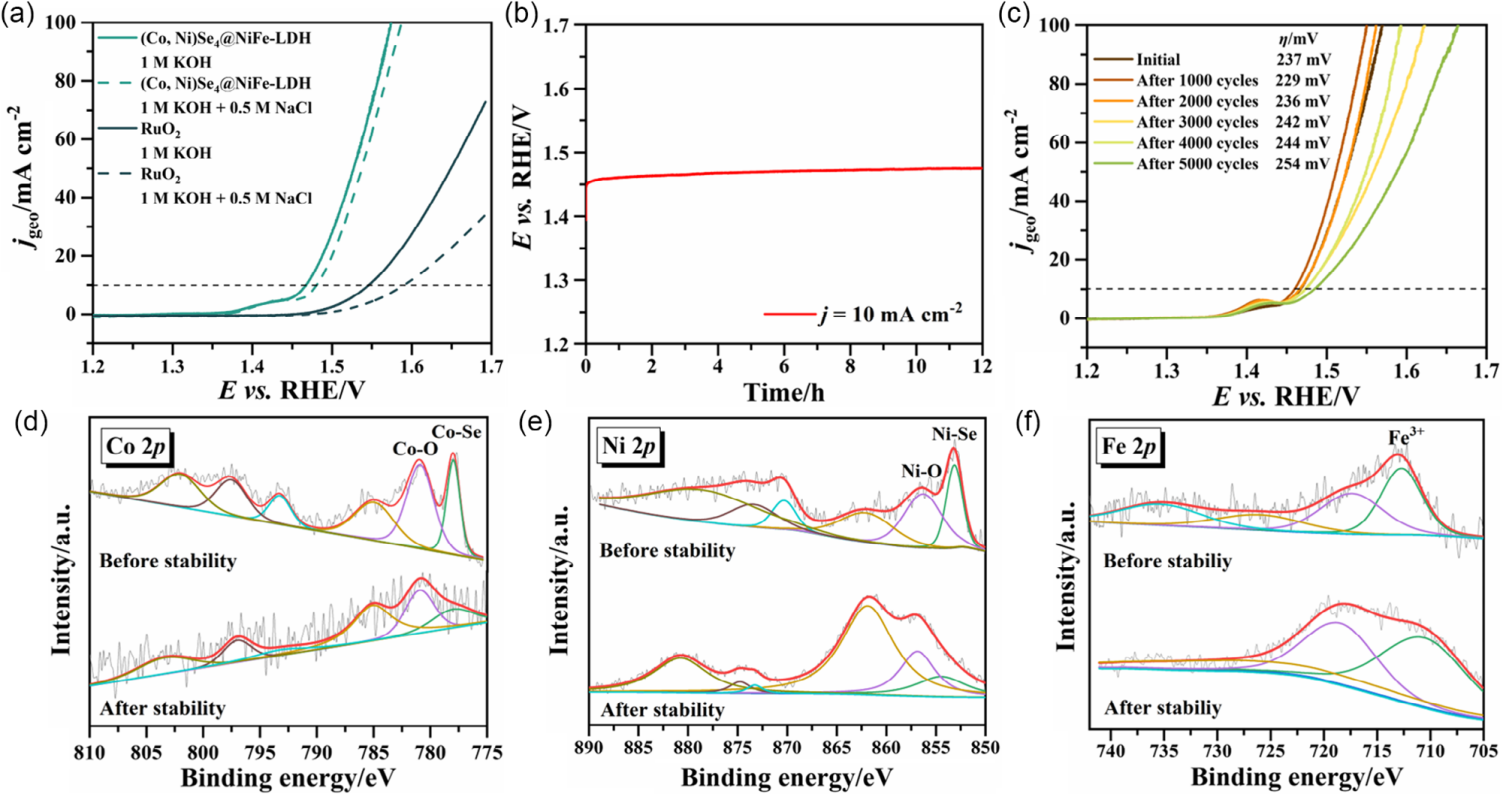
a) OER LSV curves of (Co, Ni)Se_4_@NiFe‐LDH and RuO_2_ catalysts in 1 m KOH + 0.5 m NaCl simulated seawater electrolyte; b) OER chronopotentiometry curves of the (Co, Ni)Se_4_@NiFe‐LDH at current density of 10 mA cm^−2^ in 1 M KOH solution; c) CV curves of the (Co, Ni)Se_4_@NiFe‐LDH before and after the 1000, 2000, 3000, 4000 and 5000 cycles of stability tests; XPS spectra of the (Co, Ni)Se_4_@NiFe‐LDH catalyst before and after long‐term stability tests. d) Co 2*p*; e) Ni 2*p*; and f) Fe 2*p*.

Seawater electrolysis has attracted much attention in recent years due to the problem of freshwater scarcity, but its development is still restricted by the presence of Cl^−^ (0.56576 mol kg^−1^) in seawater.^[^ ^32^ ^]^ The Cl^−^ not only diminishes the OER efficiency due to the competitive chloride oxidation reaction, but also severely corrodes the electrode.[^6^, ^33^] In this work, we further investigated the corrosion behavior of the electrocatalyst (Co, Ni)Se_4_@NiFe‐LDH against Cl^−^ by performing the OER process in artificial seawater electrolyte (1 m KOH + 0.5 m NaCl). As shown in **Figure** **6**a, the (Co, Ni)Se_4_@NiFe‐LDH shows a slight change in activity, and only requires an overpotential of 268 mV to reach a current density of 10 mA cm^−2^ in artificial seawater, which is well below the theoretical overpotential (490 mV) to trigger hypochlorite formation. In contrast, the overpotential at 10 mA cm^−2^ of commercial RuO_2_ increases from 316 to 360 mV. The above results illustrate that the (Co, Ni)Se_4_@NiFe‐LDH heterostructure possesses high resistance to Cl^−^ corrosion. Moreover, the high stability of (Co, Ni)Se_4_@NiFe‐LDH for OER is also confirmed by chronopotentiometry measurements and accelerated durability tests (ADTs) in 1 m KOH solution, cf. Figure 6b,c, respectively. After 12 h of *E–t* test at a fixed current density of 10 mA cm^−2^, the potential shows no obvious degradation. The OER activity, after 5000 cycles, shows a slight decrease with an overpotential shift of −19 mV. The chemical valence states of the electrocatalyst (Co, Ni)Se_4_@NiFe‐LDH, after the OER stability test, were studied by XPS. The XPS spectra of Co 2*p* and Ni 2*p*, after stability tests, still show the characteristic peaks of the M–O and M–Se signals, as shown in Figure 6d,e, while the intensity of the XPS peaks for the M–Se species decreases, indicating oxidation at the surface of the (Co, Ni)Se_4_@NiFe‐LDH catalyst during the OER process.^[^ ^14^ ^]^ The oxidation state of the Fe element shows no obvious change in the XPS spectrum of Fe 2*p*.

### 2.4 Theoretical Investigation of Heterostructure Engineering

**Figure 7:**
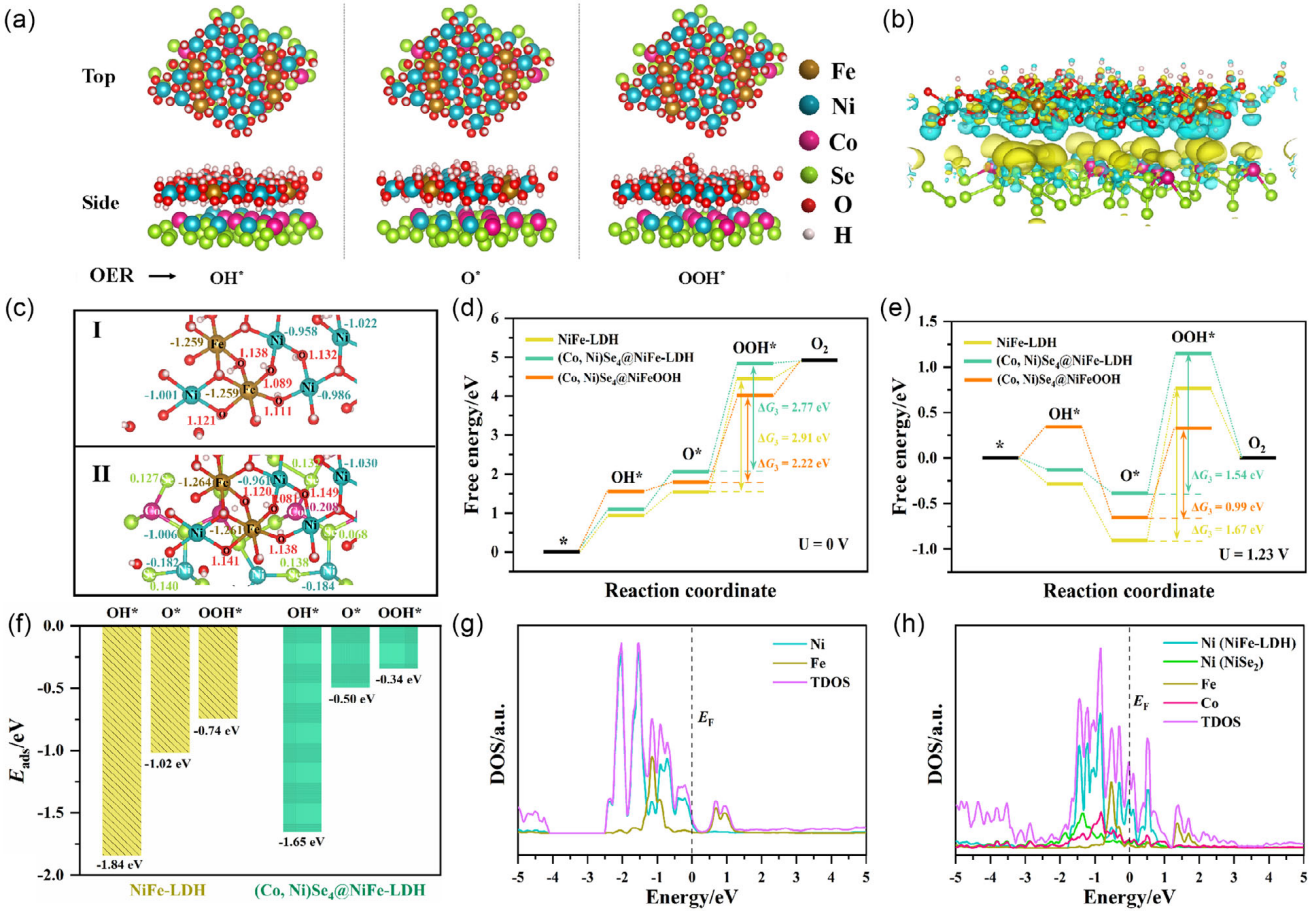
a) Top and side view of the optimized geometry of the (Co, Ni)Se_4_@NiFe‐LDH model. The corresponding geometry of OH*, O* and OOH* reaction intermediates adsorption configuration on the (Co, Ni)Se_4_@NiFe‐LDH system; b) charge density difference diagrams of the (Co, Ni)Se_4_@NiFe‐LDH system. The isovalue of the isosurface is 3.0 × 10^−3^ e Å^−3^, and the yellow and green region represent electron accumulation and depletion, respectively; c) Bader charge numbers of atoms in the (I) NiFe‐LDH and (II) (Co, Ni)Se_4_@NiFe‐LDH; Gibbs free energy diagrams of the OER processes on the NiFe‐LDH, (Co, Ni)Se_4_@NiFe‐LDH and (Co, Ni)Se_4_@NiFeOOH: d) at *U* = 0 V and e) *U* = 1.23 V; f) adsorption energy (*E*_ads_) of OH*, O*, and OOH* reaction intermediates on the (Co, Ni)Se_4_@NiFe‐LDH system; g,h) the TDOS of the NiFe‐LDH and (CoNi)Se_2_@NiFe‐LDH and the PDOS for 3*d* orbitals of Co, Ni, and Fe elements.

To further understand the interfacial interaction and synergistic effect of (Co, Ni)Se_4_ and NiFe‐LDH on the intrinsic OER activity of NiFe‐LDH, first‐principles calculations based on density functional theory (DFT) were performed. In view of the analytical results of XRD and HRTEM, i.e., the heterointerface structure and crystalline phases, an optimized heterostructure model, **Figure** **7**a, (Co, Ni)Se_4_ as core, is constructed to investigate the interfacial electronic structure and favorable energy pathway for OER catalysis in (Co, Ni)Se_4_@NiFe‐LDH, and single NiFe‐LDH is also calculated for comparison, cf. Figure S12, Supporting Information. Figure 7b shows the charge density difference of (Co, Ni)Se_4_@NiFe‐LDH. Obviously, the transferred charge is delocalized on the heterointerface, i.e., a large number of electrons are attracted to (Co, Ni)Se_4_ from NiFe‐LDH, resulting in an electron‐rich region on (Co, Ni)Se_4_ and an electron‐losing region on NiFe‐LDH. The loss of electrons from NiFe‐LDH in the heterostructure, which agrees with the XPS results, indicates that the oxidation states of Ni and Fe atoms increase and lead to higher OER activity. This phenomenon is also evident in the Bader charge analysis. In Figure 7c, for example, the average Bader charge of Ni atoms in NiFe‐LDH is −1.010|e|, whereas in the (Co, Ni)Se_4_@NiFe‐LDH heterostructure the Ni atoms in the NiFe‐LDH layer exhibit a higher electron donating capacity due to a more negative value (−1.018|e|). Thus, the interfacial interaction between (Co, Ni)Se_4_ and NiFe‐LDH has a significant effect on the electronic structure of the heterostructure.

In general, the OER process undergoes four coupled proton‐electron transfer steps. The adsorption configurations of the oxygen‐involved intermediates, including OH*, O*, and OOH*, and the Gibbs free energy (Δ*G*) profiles for the NiFe‐LDH, (Co, Ni)Se_4_@NiFe‐LDH and (Co, Ni)Se_4_@NiFeOOH systems are illustrated in Figure 7a,d,e and S13, Supporting Information, where the Ni atom in the NiFe‐LDH or NiFeOOH layer is considered to be the active site. At an electrode potential (*U*) of 0 V, the largest rise occurs in the third elementary step, i.e., the formation of OOH* (M − O* + OH^−^→ M − OOH* + e^−^), which becomes the rate‐determining step (RDS) on these three catalysts. The calculated energy changes (Δ*G*_3_) of the RDS in NiFe‐LDH, (Co, Ni)Se_4_@NiFe‐LDH and (Co, Ni)Se_4_@NiFeOOH were 2.91, 2.77 and 2.22 eV, respectively, implying that the (Co, Ni)Se_4_@NiFe‐LDH and (Co, Ni)Se_4_@NiFeOOH heterostructures possess faster OER kinetics. Similarly, at *U* = 1.23 V, the energy barrier for OOH* formation is 1.54 eV for (Co, Ni)Se_4_@NiFe‐LDH and 0.99 eV for (Co, Ni)Se_4_@NiFeOOH, which is much lower than that for NiFe‐LDH (1.67 eV). Moreover, pure NiFe‐LDH shows a more negative free energy, suggesting more robust interactions between active sites and intermediates, which is also reflected in its more negative adsorption energies of OH*, O*, and OOH* intermediates, Figure 7f. It is well‐known that too strong or weak interaction of reactants and intermediates in electrocatalysts can negatively affect the catalytic activity.^[^ ^34^ ^]^ In a word, the above results highlight that heterostructure engineering can significantly lower the energy barrier of the RDS by modulating the electronic structure, resulting in more favorable reaction kinetics for OER. Moreover, the electrical conductivity of an electrocatalyst also plays a key role in the catalytic activity, which can be evaluated by the total density of states (TDOS) and partial density of states (PDOS), cf. Figure 7g,h. It can be observed that the (Co, Ni)Se_4_@NiFe‐LDH heterostructure presents a metallic feature due to the continuous TDOS around the Fermi level, while NiFe‐LDH presents a semiconducting feature. Moreover, compared to NiFe‐LDH, the TDOS of the (Co, Ni)Se_4_@NiFe‐LDH exhibits higher electron density near the Fermi level, suggesting enhanced conductivity and increased carrier concentration during the OER, which can be attributed to the synergistic effect at the heterointerface.^[^ ^35^ ^]^ PDOS demonstrates that the 3*d* orbital of Ni site in the NiFe‐LDH layer is responsible for the enhanced electronic DOS near the Fermi level of the (Co, Ni)Se_4_@NiFe‐LDH, manifesting that the active Ni sites possess enhanced electron‐transport capacity for OER. Overall, the higher OER performance of the (Co, Ni)Se_4_@NiFe‐LDH heterostructure compared to its counterpart (NiFe‐LDH) can be attributed to the following aspects: 1) the lowering of the RDS energy barrier, i.e., the formation of OOH* intermediate, which accelerates the OER kinetics; 2) the optimized adsorption strength of the intermediates on Ni active sites; 3) the modulated electronic structure, which results in an increase in the oxidation states of Ni and Fe and thus enhances the OER activity; and 4) the improved conductivity. Therefore, reasonable interface engineering with strong electron interaction can optimize the adsorption free energies of intermediates and lower the energy barrier of RDS, thus accelerating the OER electrocatalytic process.

## 3 Conclusion

In summary, a (Co, Ni)Se_4_@NiFe‐LDH heterostructure catalyst was designed and developed. The (Co, Ni)Se_4_@NiFe‐LDH heterostructure shows significantly superior OER performance compared to the individual (Co, Ni)Se_4_ and NiFe‐LDH catalysts. From the experimental results and theoretical calculations, it is identified that Ni atoms are catalytically active sites, and their catalytic activity is modulated by the electron density. The strong electron interaction at the interface leads to electron redistribution. As a result, the valence states of Ni and Fe increase, which can achieve higher catalytic activity of OER. In addition, the modulation of electronic structure results in a lower energy barrier of OOH* intermediate formation (RDS) and proper adsorption strength of intermediates on Ni active sites, thus improving the kinetics of OER. This work can serve as a guide to design excellent electrocatalysts for water splitting by constructing heterostructure.

## Conflict of Interest

The authors declare no conflict of interest.

## Supporting information

Supplementary Material

## Data Availability

The data that support the findings of this study are available from the corresponding author upon reasonable request.
