# Supplementary material for: Heterostructures by Templated Synthesis of Layered Double Hydroxide to Modulate the Electronic Structure of Nickel Sites for a Highly Efficient Oxygen Evolution Reaction
Source: Small Sci. 2024 Feb 28;4(4):2300294. doi: 10.1002/smsc.202300294 (PMC11935000; doi:10.1002/smsc.202300294)
Supplement: Supplementary file 1 — Supplementary Material [file SMSC-4-2300294-s001.pdf]

Supporting Information

Heterostructures by Templated Synthesis of Layered Double Hydroxide to Modulate the Electronic Structure of Nickel Sites for a Highly Efficient Oxygen Evolution Reaction

Kexin Xu[a], Haihong Zhong[b,*], Xuying Li[a], Jie Song[a], Luis Alberto Estudillo-Wong[e], Jun Yang[c], Yongjun Feng[a,*], Xiaojuan Zhao[d,*], Nicolas Alonso-Vante[f,*]

[a] *State Key Laboratory of Chemical Resource Engineering, Beijing Engineering Center for Hierarchical Catalysts, College of Chemistry, Beijing University of Chemical Technology, No. 15 Beisanhuan East Road, Beijing 100029, China.*

[b] *School of Chemistry and Chemical Engineering, Hainan University, No. 58 Renmin Road, Haikou 570228, P. R. China.*

[c] *State Key Laboratory of Multiphase Complex Systems, Institute of Process Engineering, Chinese Academy of Sciences, Beijing 100190, China.*

[d] *Institute of High Energy Physics, Chinese Academy of Science, Beijing 100190, China.*

[e] *Departamento de Biocienciase Ingeniería, CIIEMAD-IPN, Instituto Politécnico Nacional, C.P. 07340 Ciudad de México, México*

[f] *IC2MP, UMR-CNRS 7285, University of Poitiers, Poitiers Cedex F-86022, France.*

*Correspondence: hzhong@hainanu.edu.cn (H. Zhong); yjfeng@mail.buct.edu.cn (Y. Feng); zhaoxiaojuan@ihep.ac.cn (X. Zhao); nicolas.alonso.vante@univ-poitiers.fr (NAV)

1. EXPERIMENTAL SECTION

1.1 Materials. Cobaltous nitrate hexahydrate ($Co(NO_3)_2·6H_2O$, AR, Shanghai Macklin Biochemical Co., Ltd.), nickel nitrate hexahydrate ($Ni(NO_3)_2·6H_2O$, AR, Shanghai Macklin Biochemical Co., Ltd.), aluminum nitrate nonahydrate ($Al(NO_3)_3·9H_2O$, AR, Shanghai Macklin Biochemical Co., Ltd.), ferric nitrate nonahydrate ($Fe(NO_3)_3·9H_2O$, AR, Shanghai Macklin Biochemical Co., Ltd.), hexamethylenetetramine ($C_6H_{12}N_4$, AR, Shanghai Macklin Biochemical Co., Ltd.), urea ($CO(NH_2)_2$, AR, Shanghai Macklin Biochemical Co., Ltd.), 3-Aminobenzene sulfonic acid ($C_6H_7NO_3S$, AR, Shanghai Macklin Biochemical Co., Ltd.), ammonium fluoride (NH_4F, AR, Shanghai Macklin Biochemical Co., Ltd.), sodium hydroxide (NaOH, AR, Shanghai Macklin Biochemical Co., Ltd.), selenium powder (Se, AR, Shanghai Macklin Biochemical Co., Ltd.), active carbon XC-72 (AR, Shanghai Cabot Chemical Co., Ltd.), and commercial ruthenium dioxide (RuO_2, Shanghai Macklin Biochemical Co., Ltd.) were used without further purification. The deionized water with electrical conductance less than 10^{-6} $S·cm^{-2}$ was obtained from the Milli-Q system.

1.2 Catalysts Preparation.

1.2.1 Synthesis of $(CoNi)_3Al_1$-MA-LDH precursor. The metanilic acid intercalated $(CoNi)_3Al_1$-MA-LDH flower-like precursor was synthesized via a facile hydrothermal reaction. Typically, $Co(NO_3)_3·6H_2O$ (0.4366 g, 1.5 mmol), $Ni(NO_3)_3·6H_2O$ (0.4362 g, 1.5 mmol), $Al(NO_3)_3·9H_2O$ (0.3751 g, 1 mmol), NH_4F (0.1482 g, 4 mmol), hexamethylenetetramine (0.7010 g, 5 mmol) were completely dissolved into 70 mL of deionized water and kept stirring for 15 min to form a transparent solution A. Meanwhile, 1 M NaOH solution was dropwise added to the metanilic acid (1.7319 g, 10 mmol) until pH = 7.0 to form a solution B. Then, the solution A and solution B were rapidly mixed under magnetic stirring and stirred for another 30 min. Subsequently, the mixed solution was transferred into a 100 mL Teflon-lined stainless-steel autoclave and kept at 100°C for 12 h. Finally, the $(CoNi)_3Al_1$-MA-LDH precursor was collected, washed with deionized water and ethanol for three times, and dried in vacuum for 10 h.

1.2.2 Synthesis of the (Co, Ni)Se_4 catalyst. Firstly, the $(CoNi)_3Al_1$-MA-LDHs precursor was subjected to carbonization at 500°C with a heating rate of 3°C/min for 1 h under a nitrogen

atmosphere. The product was added to 2 M NaOH solution and kept stirring for 1 h. Then, the suspension was washed with deionized water and ethanol until pH ≈ 7, and the obtained product was donated as e-LDHs. Se powder and e-LDHs ($m_{Se\ powder}/m_{e\text{-}LDHs}$ = 1:2) were fully ground for 15 min, and then subjected to the selenylation treatment at 400°C under N_2 atmosphere for 2 h with a heating rate of 1°C min^{-1}. Finally, the obtained product was named as (Co, Ni)Se_4.

1.2.3 Synthesis of the (Co, Ni)Se_4@NiFe-LDH and NiFe-LDH catalysts. Typically, $Ni(NO_3)_3\cdot 6H_2O$ (0.4362 g, 1.5 mmol), $Fe(NO_3)_3\cdot 9H_2O$ (0.2020 g, 0.5 mmol) and $CO(NH_2)_2$ (0.3000 g, 5 mmol) were successively added into 50 mL of deionized water under magnetic stirring for 10 min in order to form a homogeneous light-brown solution. Subsequently, 0.1000 g of as-prepared (Co, Ni)Se_4 powder was added into the above solution and stirred for 1 h. Then, the resulting solution was poured into a Teflon-lined stainless-steel autoclave (100 mL) and kept at 120°C in an oven for 10 h. Finally, the precipitate was washed with deionized water and ethanol, then dried at 60°C overnight. The sample was donated as (Co, Ni)Se_4@NiFe-LDH. By adjusting the selenide/LDH or Ni/Fe ratio, a series of (Co, Ni)Se_4@NiFe-LDH samples were prepared. Besides, the synthesis procedure of NiFe-LDH is the same as the proposal of the (Co, Ni)Se_4@NiFe-LDH but without the addition of as-prepared (Co, Ni)Se_4 powder.

1.2.4 *Synthesis of the (Co, Ni)Se_4 + NiFe-LDH physical mixture (PM) catalyst.* The physical mixture of (Co, Ni)Se_4 + NiFe-LDH was prepared simply by mechanical mixing. Specifically, 0.1000 g of prepared (Co, Ni)Se_4 powder and the obtained NiFe-LDH powder were ground in the quartz mortar until well mixed.

1.3 Physicochemical Characterization. The crystal structure and chemical composition of the samples were analyzed via a Shimadzu XRD-6000 X-ray diffractometer using Cu-Kα (λ = 0.15406 nm) radiation at 40 kV and 30 mA. The morphology of the samples was investigated by a Zeiss Supra 55 scanning electron microscope (SEM). The high-resolution transmission electron microscope (HRTEM) was carried out at the JEOL JEM-2100F operating at 200 kV. Fourier transform infrared (FT-IR) spectra were collected by a Vector 22 (Bruker) spectrophotometer using the KBr pellet technique from 400 to 4000 cm^{-1} with a resolution of 2 cm^{-1}. Based on Van der Pauw's

method, the electrical properties of the catalysts were evaluated using a Phystech RH2035 Hall measurement system at room temperature with a magnetic field of 0.45 T. XPS spectra were collected by VG ESCA-LAB 2201 XL spectrometer with an Al-Kα anode. The calibration peak was C 1*s* at 284.4 eV. Nitrogen adsorption-desorption isotherms were obtained by the Brunauer-Emmett-Teller (BET) method, and the pore size and volume were determined by the Barrett-Joyner-Halenda (BJH) measurement using a BSD-PM (Beishide Instrument-S&T. (Beijing) CO., Ltd.). The metal (Co, Ni, and Fe) L-edge X-ray absorption spectra were collected at room temperature at beamline 4B7B of the Beijing Synchrotron Radiation Facility (BSRF, operated at 2.5 GeV with a maximum current of 250 mA), using a grating monochromator. The spectra of the sample A were measured in total-electronic-yield mode. The K-edge X-ray absorption fine structure spectra were collected at 1W1B station in Beijing Synchrotron Radiation Facility (BSRF). The storage rings of BSRF were operated at 2.5 GeV with an average current of 250 mA. Using Si (111) double-crystal monochromator, the data collection was carried out in transmission/fluorescence mode using the ionization chamber. All spectra were collected in ambient conditions. In situ Raman spectra were collected at LabRAM HR Evol (Horiba, Confocal Raman Microscopy) with the laser of 633 nm (ND Filter: 5%, Acq. Time1: 10 s, Accumulat:5, Acq. Time2: 56 s).

1.4 Electrochemical measurements. The electrochemical tests were carried out on a DH7000 Jiangsu Donghua Analysis Instruments Co., Ltd. with a standard three-electrode system. All applied electrode potential was converted to a reversible hydrogen electrode (RHE) using the equation ($E_{(RHE)}$/V = $E_{(Hg/HgO)}$ + 0.059 × pH + $E^{0}_{Hg/HgO}$, where $E^{0}_{Hg/HgO}$ is 0.098 V in 1 M KOH at 25°C). The electrolyte was the 1 M KOH solution. A glassy carbon (GC) electrode (0.196 cm^{-2}), a platinum wire and an Hg/HgO electrode were used as the working electrode, the counter electrode, and the reference electrode, respectively. For the preparation of the ink, 3.0 mg of the catalyst, 7.0 mg of carbon black and 50 μL of Nafion solution (5.0 wt%) were dispersed in a solution of ethanol (900 μL) and deionized water (50 μL) under sonication for 2 h. Then, 16.6 μL of the ink was dropped onto the surface of the GC electrode, and the mass loading of the catalyst is 0.25 mg cm^{-2}.

The cyclic voltammograms (CVs) were recorded from 1.0-1.7 V *vs.* RHE with a scan rate of 50 mV s^{-1} in Ar-saturated or O_2-saturated 1 M KOH solution. The linear sweep voltammograms were collected from 1.2 to 1.7 V *vs.* RHE with a scan rate of 5 mV s^{-1} at 1600 rpm with 85% *iR* compensation. The Tafel slope was calculated by the equation: η = blog(j)+a, where η, b, and j are the overpotential, Tafel slope, and current density, respectively. The seawater splitting activity was evaluated in the 1 M KOH + 0.5 M NaCl electrolyte. The electrochemical double-layer capacitance (C_{dl}) was measured by using cyclic voltammetry at different scan rates (20-120 mV s^{-1}) in the non-faradic potential region of 1.1-1.2 V *vs.* RHE to evaluate the electrochemically active surface area (ECSA). The ECSA values were calculated by the following equation: ECSA = C_{dl}/C_s, where C_s is determined to be 0.04 mF cm^{-2} in 1 M KOH electrolyte. The ECSA-normalized current density was estimated by the following equation：$j_{ECSA} = j_{geo}$/ECSA, where j_{geo} represents the geometric current density. The electrochemical impedance spectrum (EIS) was obtained at open circuit voltage between 100 kHz and 0.1 Hz with an amplitude of 5 mV. Chronoamperometric measurements were performed for 12 h at the corresponding potential required to achieve the current density of 10 mA cm^{-2} and the accelerated durability test (ADT) were carried out at 1600 rpm in the 1 M KOH solution.

1.5 DFT calculations. All the calculations were performed in the framework of the density functional theory with the projector augmented plane-wave method, as implemented in the Vienna ab initio simulation package [1]. The generalized gradient approximation proposed by Perdew, Burke, and Ernzerhof was selected for the exchange-correlation potential [2]. The cut-off energy for plane wave was set to 400 eV. The energy criterion was set to 10 − 5 eV in iterative solution of the Kohn-Sham equation. A vacuum layer of 15 Å was added perpendicular to the sheet to avoid artificial interaction between periodic images. The Brillouin zone integration was performed using a 2×2×1 k-mesh. All the structures were relaxed until the residual forces on the atoms have declined to less than 0.03 eV/Å.

The adsorption free energy was defined as:

$$\Delta G_H = \Delta E + \Delta E_{ZPE} - T\Delta S$$

where the ΔE is the adsorption energy on the cluster surface from DFT calculations. The ΔE_{ZPE} and ΔS are the difference for the zero-point energy and entropy, respectively. The zero-point energy and entropy were calculated at the standard conditions corresponding to the pressure of 101325 Pa (~1 bar) of H_2 at the temperature of 298.15 K.

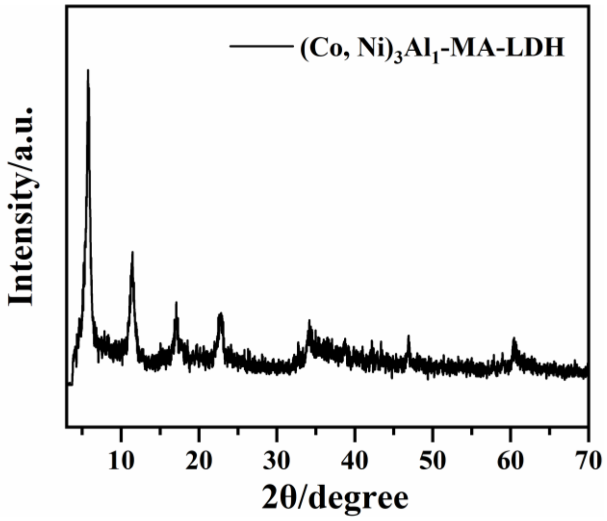

Figure S1. XRD pattern of the (Co, Ni)$_3$Al$_1$-MA-LDH precursor.

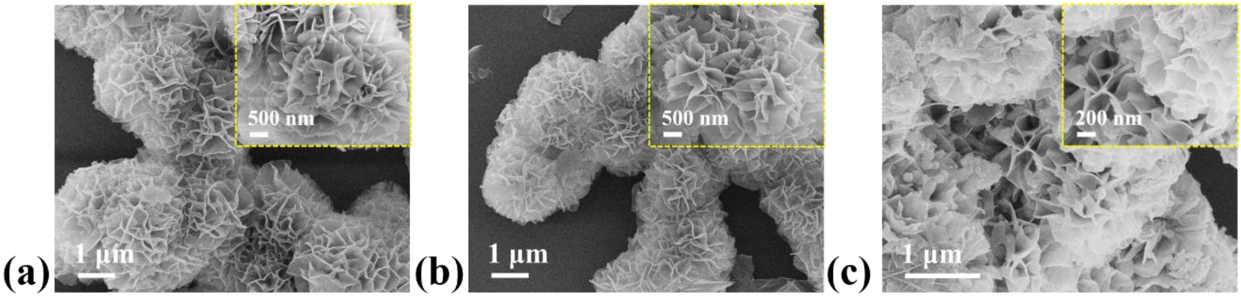

Figure S2. (a-c) SEM images of (Co, Ni)$_3$Al$_1$-MA-LDH, e-LDH and (Co, Ni)Se$_4$ samples, respectively.

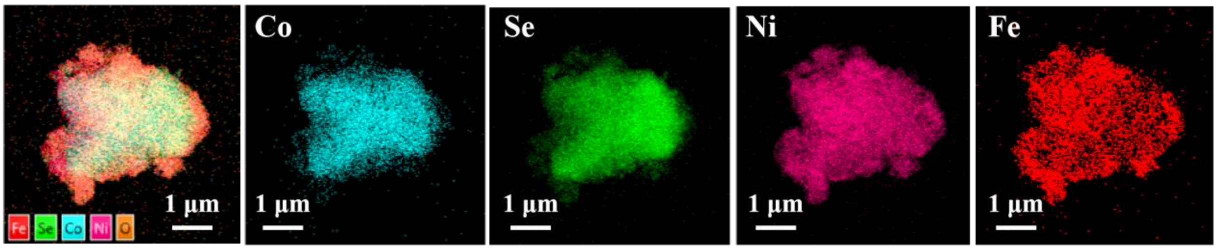

Figure S3. Elemental mapping images of the (Co, Ni)Se$_4$@NiFe-LDH.

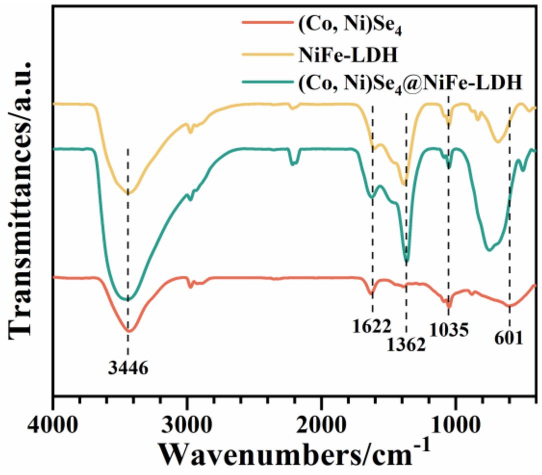

Figure S4. FT-IR spectra of (Co, Ni)Se_4, NiFe-LDH, and (Co, Ni)Se_4@NiFe-LDH samples.

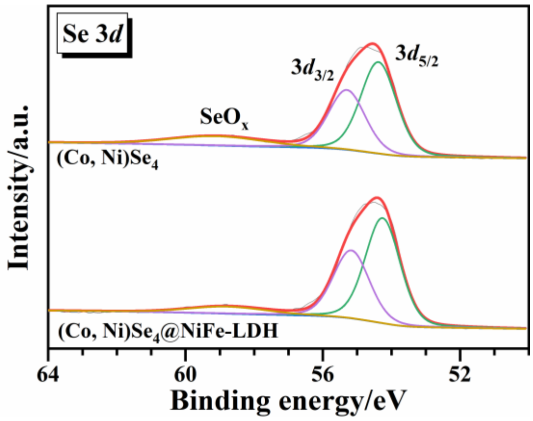

Figure S5. XPS spectra of Se 3*d* orbital for (Co, Ni)Se_4 and (Co, Ni)Se_4@NiFe-LDH samples.

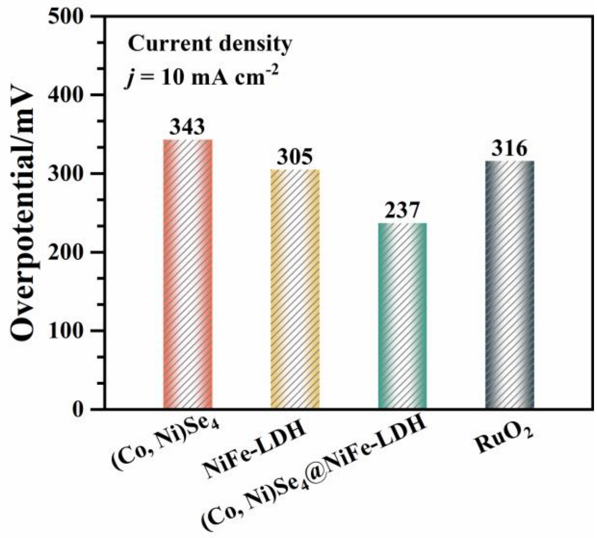

Figure S6. Overpotential comparison of the (Co, Ni)Se_4, NiFe-LDH, (Co, Ni)Se_4@NiFe-LDH and RuO_2 catalysts at 10 mA cm^{-2} in 1 M KOH solution.

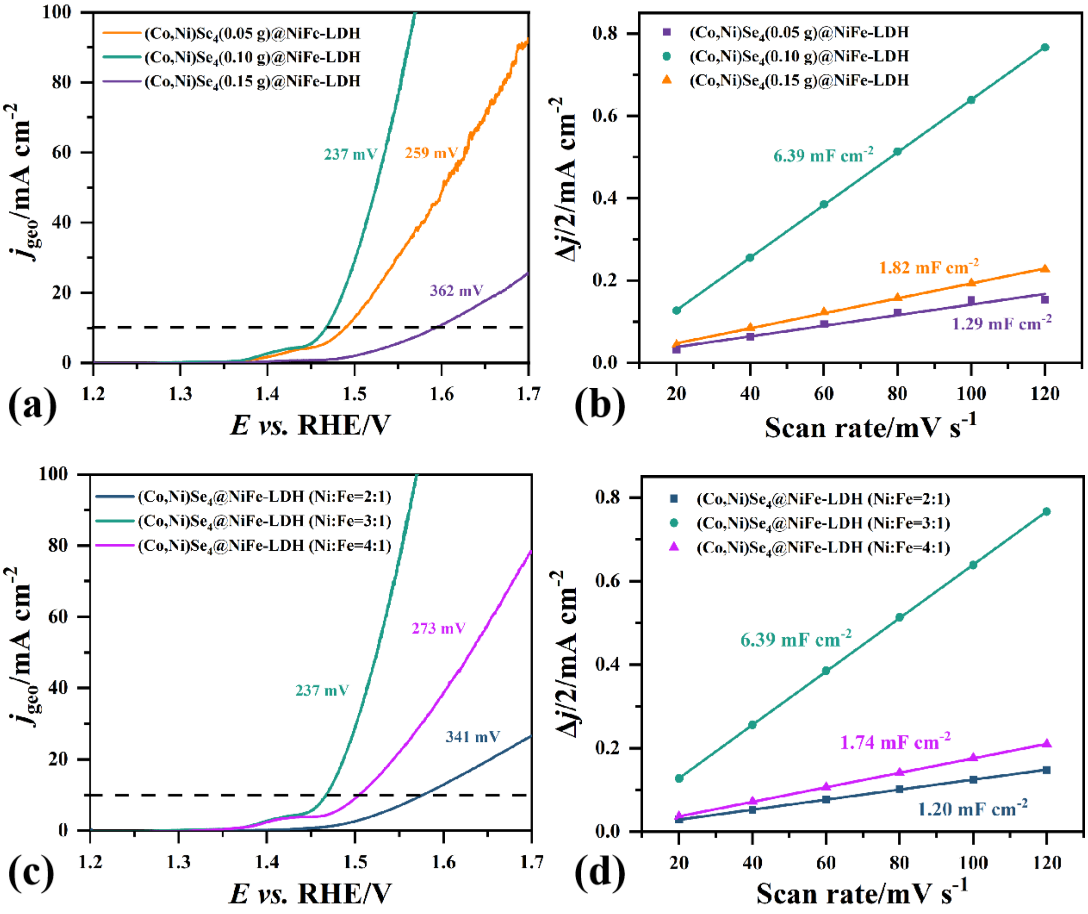

Figure S7. LSV polarization curves and double-layer capacitance (C_{dl}) plots of (Co, Ni)Se_4@NiFe-LDH catalysts with different selenide/LDH or Ni/Fe ratio in 1 M KOH solution.

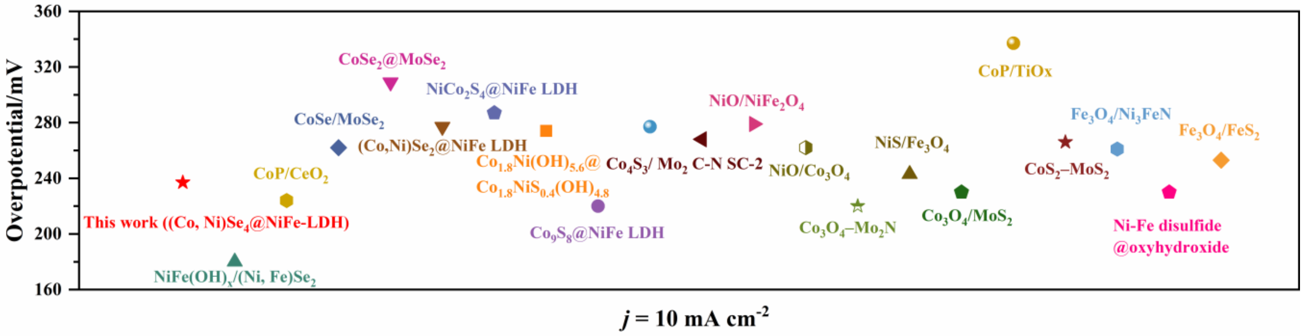

Figure S8. Overpotential comparison with other reported transition metal-based heterostructure electrocatalysts for OER at 10 mA cm^{-2}.

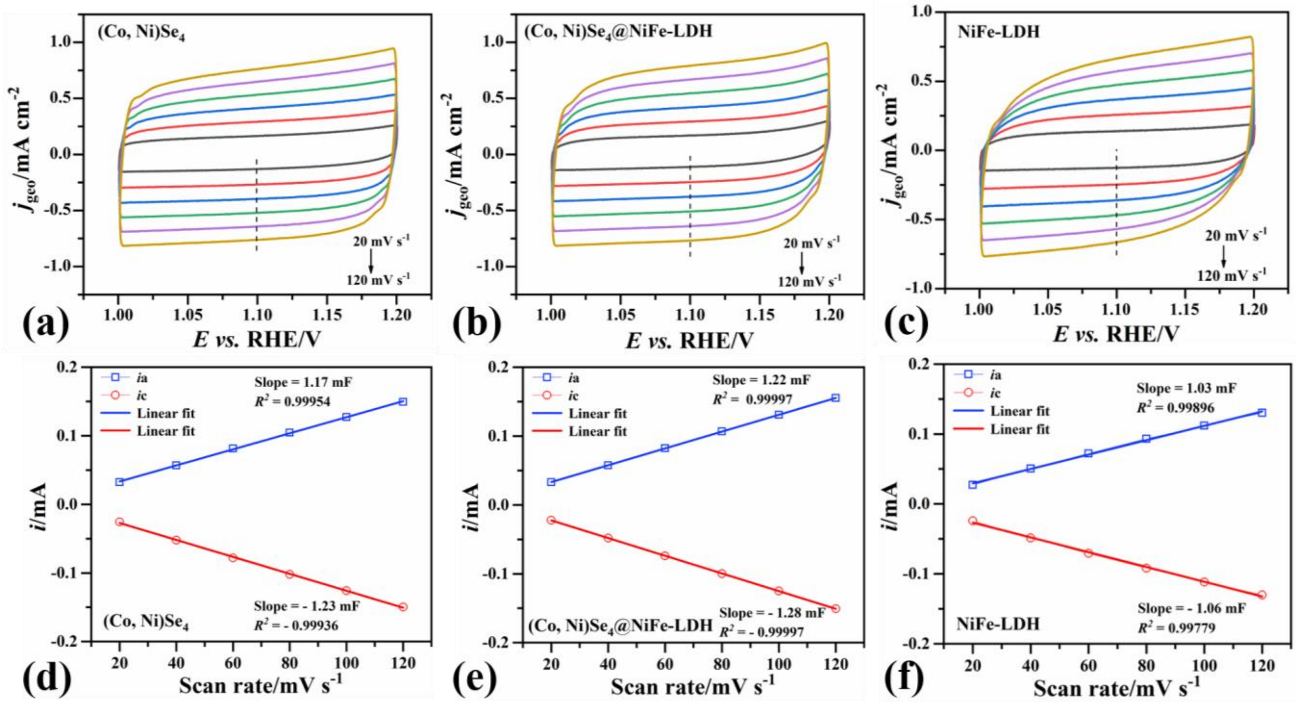

Figure S9. ECSA measurements for all the samples at different scan rates from 20 to 120 mV s^{-1} in N_2-saturated 1 M KOH within a non-faradaic region of 1.0-1.2 V *vs.* RHE.

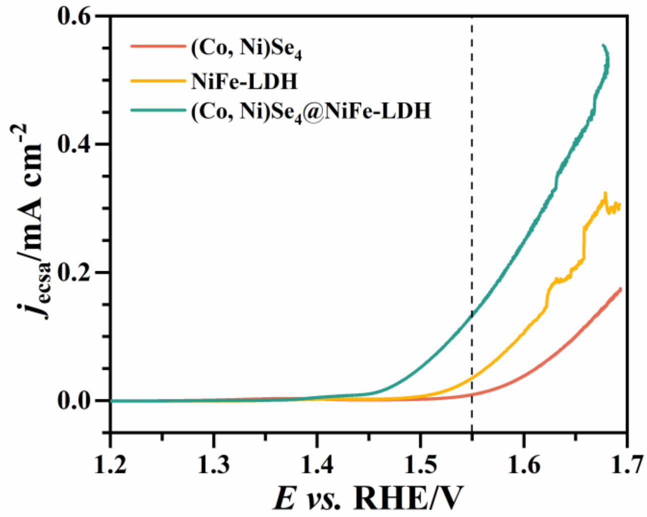

Figure S10. ECSA-normalized LSV curves of the (Co, Ni)Se_4, NiFe-LDH, and (Co, Ni)Se_4@NiFe-LDH catalysts.

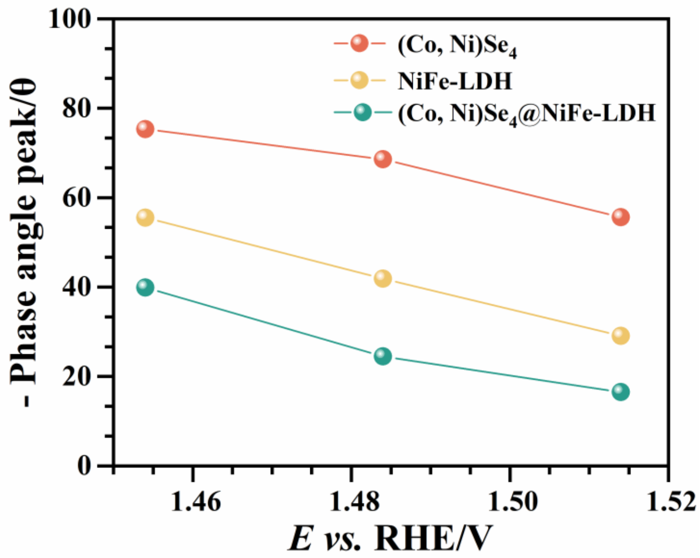

Figure S11. Phase angles of the (Co, Ni)Se_4, NiFe-LDH, and (Co, Ni)Se_4@NiFe-LDH catalysts at the potential range of 1.454-1.514 V *vs.* RHE.

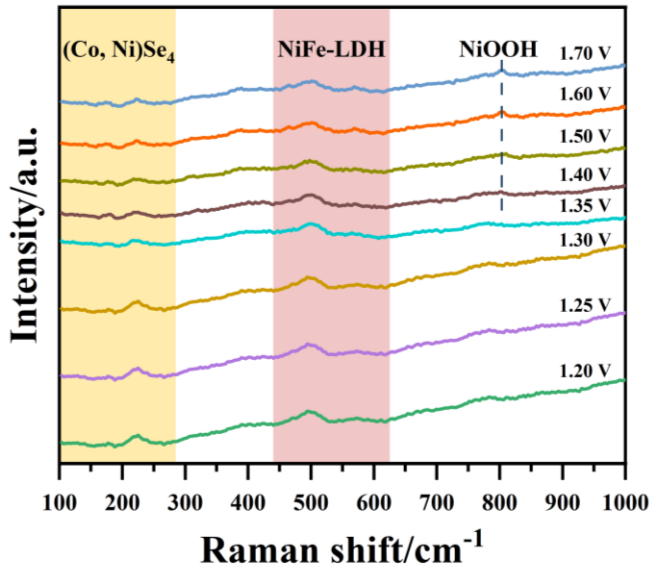

Figure S12. In situ Raman spectra of the (Co, Ni)Se_4@NiFe-LDH tested at various potentials.

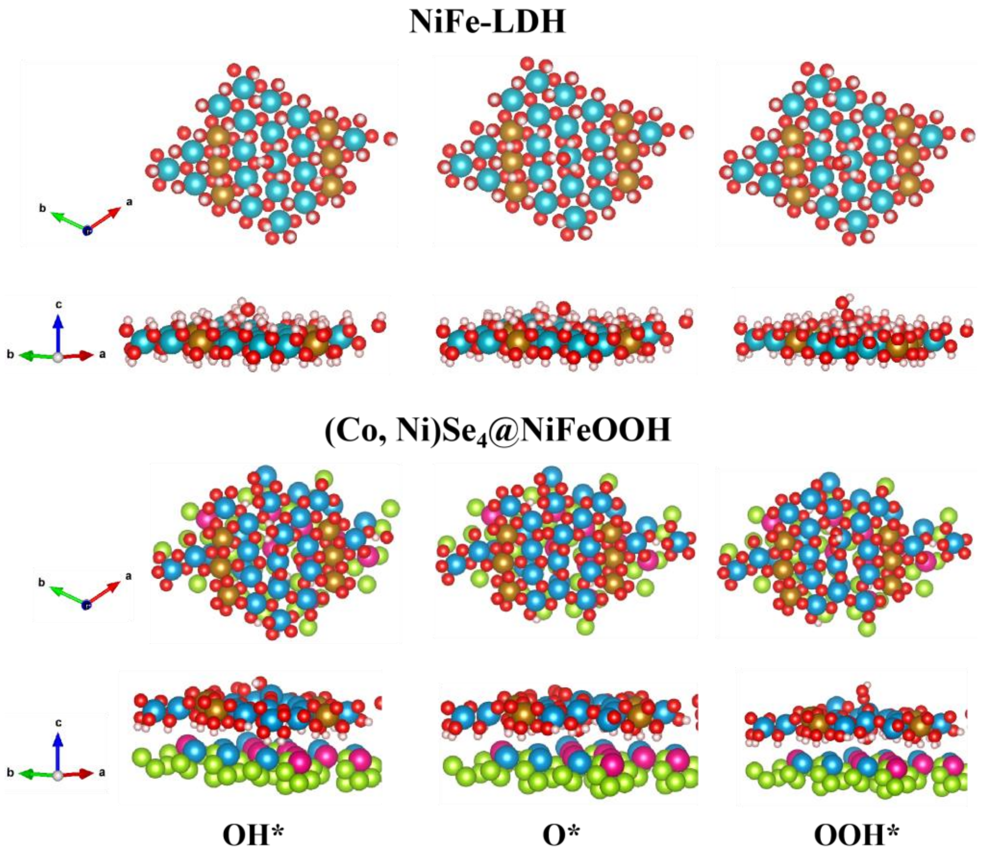

Figure S13. Top and side view of the optimized geometry of the NiFe-LDH and (Co, Ni)Se_4@NiFeOOH models. The corresponding geometry of OH*, O* and OOH* reaction intermediates adsorption configuration on the systems.

Table S1. EXAFS fitting parameters at the Co K-edge for various samples.

Sample	Shell	N [a]	R (Å) [b]	σ^2 (Å^2·10^{-3}) [c]	ΔE_0 (eV) [d]	*R* factor (%)
(Co, Ni)Se_4	Co-Se	4.1	2.14	6.3	6.2	0.8
(Co, Ni)Se_4@NiFe-LDH	Co-Se	3.7	2.10	6.0	5.4	0.8

[a] *N*: coordination numbers; [b] *R*: bond distance; [c] σ^2: Debye-Waller factors; [d] ΔE_0: the inner potential correction. *R* factor: goodness of fit. *S*02 was set as 1 for Fe-O/Fe-O-Fe which was obtained from the experimental EXAFS fit of reference $CoSe_2$ by fixing CN as the known crystallographic value and was fixed to all the samples.

Table S2. EXAFS fitting parameters at the Ni K-edge for various samples.

Sample	Shell	N [a]	R (Å) [b]	σ^2 (Å^2·10^{-3}) [c]	ΔE_0 (eV) [d]	*R* factor (%)
(Co, Ni)Se_4	Ni-Se	4.6	2.19	7.1	6.4	0.9
(Co, Ni)Se_4@NiFe-LDH	Ni-O	4.8	1.60	7.3	0.3	1.0
	Ni-O-Ni	4.6	2.69	8.7	-5.4	
NiFe-LDH	Ni-O	5.4	1.58	7.4	-3.1	0.6
	Ni-O-Ni	4.0	2.71	9.6	-5.0	

[a] *N*: coordination numbers; [b] *R*: bond distance; [c] σ^2: Debye-Waller factors; [d] ΔE_0: the inner potential correction. *R* factor: goodness of fit. *S*02 was set as 0.88/0.94 for Ni-O/Ni-O-Ni which was obtained from the experimental EXAFS fit of reference NiO by fixing CN as the known crystallographic value and was fixed to all the samples.

Table S3. EXAFS fitting parameters at the Fe K-edge for various samples.

Sample	Shell	N [a]	R (Å) [b]	σ^2 (Å^2·10^{-3}) [c]	ΔE_0 (eV) [d]	*R* factor (%)
(Co, Ni)Se_4@NiFe-LDH	Fe-O	5.7	1.54	5.8	-3.0	0.4
	Fe-O-Fe	4.4	2.72	7.8	-4.1	
	Fe-O-Fe	1.4	3.13	10.1		
NiFe-LDH	Fe-O	5.5	1.51	9.8	-2.2	0.6
	Fe-O-Fe	4.3	2.69	9.9	-2.7	
	Fe-O-Fe	1.6	3.20	10.2		

[a] *N*: coordination numbers; [b] *R*: bond distance; [c] σ^2: Debye-Waller factors; [d] ΔE_0: the inner potential correction. *R* factor: goodness of fit. *S*02 was set as 0.85/0.92 for Fe-O/Fe-O-Fe which was obtained from the experimental EXAFS fit of reference RuO_2 by fixing CN as the known crystallographic value and was fixed to all the samples.

Table S4. EXAFS fitting parameters at the Se K-edge for various samples.

Sample	Shell	N [a]	R (Å) [b]	σ^2 (Å^2·10^{-3}) [c]	ΔE_0 (eV) [d]	*R* factor (%)
(Co, Ni)Se_4	Se-Co	2.0	2.42	3.8	4.7	1.0
(Co, Ni)Se_4@NiFe-LDH	Se-Co	1.9	2.44	4.0	6.2	1.2

[a] *N*: coordination numbers; [b] *R*: bond distance; [c] σ^2: Debye-Waller factors; [d] ΔE_0: the inner potential correction. *R* factor: goodness of fit. *S*02 was set as 1 for Se-Co which was obtained from the experimental EXAFS fit of reference $CoSe_2$ by fixing CN as the known crystallographic value and was fixed to all the samples.

Table S5. Comparison of the overpotential and Tafel slope for the OER on the (Co, Ni)Se_4@NiFe-LDH catalyst and other heterostructured electrocatalysts reported in the literature in alkaline media.

Electrocatalysts	Overpotential/mV @10 mA cm^{-2}	Tafel slope/mV dec^{-1}	Refs.
**(Co, Ni)Se_4@NiFe-LDH**	**237**	**65.26**	**This work**
NiFe$(OH)_x$/(Ni, Fe)Se_2	180	42	[3]
CoP/CeO_2	224	90.3	[4]
CoSe/$MoSe_2$	262	54.9	[5]
$CoSe_2$@$MoSe_2$	309	84.04	[6]
(Co, Ni)Se_2@NiFe LDH	277	75	[7]
$NiCo_2S_4$@NiFe LDH	287	86.4	[8]
$Co_{1.8}Ni(OH)_{5.6}$@$Co_{1.8}NiS_{0.4}(OH)_{4.8}$	274	45	[9]
Co_9S_8@NiFe LDH	220	52	[10]
Ni_3N-NiMoN	277	118	[11]
Co_4S_3/ Mo_2 C-N SC-2	268	61.2	[12]
NiO/$NiFe_2O_4$	279	42	[13]
NiO/Co_3O_4	262	58	[14]
Co_3O_4–Mo_2N	220	87.8	[15]
NiS/Fe_3O_4	243	44.2	[16]
Co_3O_4/MoS_2	230	45	[17]
CoP/TiO_x	337	72.1	[18]
CoS_2–MoS_2	266	104	[19]
Fe_3O_4/Ni_3FeN	261	43.6	[20]
Ni-Fe disulfide@oxyhydroxide	230	42.6	[21]
Fe_3O_4/FeS_2	253	48	[22]

References

[1] G. Kresse, D. Joubert, *Phys. Rev. B* **1999**, *59*, 1758-1775.

[2] J. P. Perdew, K. Burke, M. Ernzerhof, *Phys. Rev. Lett.* **1996**, *77*, 3865-3868.

[3] C. Liu, Y. Han, L. Yao, L. Liang, J. He, Q. Hao, J. Zhang, Y. Li, H. Liu, *Small* **2021**, *17*, 2007334.

[4] M. Li, X. Pan, M. Jiang, Y. Zhang, Y. Tang, G. Fu, *Chem. Eng. J.* **2020**, *395*, 125160.

[5] M. Yuan, S. Dipazir, M. Wang, Y. Sun, D. Gao, Y. Bai, M. Zhang, P. Lu, H. He, X. Zhu, S. Li, Z. Liu, Z. Luo, G. Zhang, *J. Mater. Chem. A* **2019**, *7*, 3317-3326.

[6] M. Qin, S. Fan, L. Wang, G. Gan, X. Wang, J. Cheng, Z. Hao, X. Li, *J. Colloid Interface Sci.* **2020**, *562*, 540-549.

[7] J. G. Li, H. Sun, L. Lv, Z. Li, X. Ao, C. Xu, Y. Li, C. Wang, *ACS Appl. Mater. Interfaces* **2019**, *11*, 8106-8114.

[8] X. Feng, Q. Jiao, W. Chen, Y. Dang, Z. Dai, S. L. Suib, J. Zhang, Y. Zhao, H. Li, C. Feng, *Appl. Catal. B: Environ* **2021**, *286*, 119869.

[9] B. Wang, C. Tang, H. F. Wang, X. Chen, R. Cao, Q. Zhang, *Adv. Mater.* **2019**, *31*, 1805658.

[10] X. Feng, Q. Jiao, Z. Dai, Y. Dang, S. L. Suib, J. Zhang, Y. Zhao, H. Li, C. Feng, A. Li, *J. Mater. Chem. A* **2021**, *9*, 12244-12254.

[11] A. Wu, Y. Xie, H. Ma, C. Tian, Y. Gu, H. Yan, X. Zhang, G. Yang, H. Fu, *Nano Energy* **2018**, *44*, 353-363.

[12] Y. Liu, Y. Chen, Y. Tian, T. Sakthivel, H. Liu, S. Guo, H. Zeng, Z. Dai, *Adv. Mater.* **2022**, *34*, 2203615.

[13] H. Zhong, G. Gao, X. Wang, H. Wu, S. Shen, W. Zuo, G. Cai, G. Wei, Y. Shi, D. Fu, C. Jiang, L. W. Wang, F. Ren, *Small* **2021**, *17*, 2103501.

[14] J. Zhang, J. Qian, J. Ran, P. Xi, L. Yang, D. Gao, *ACS Catal.* **2020**, *10*, 12376-12384.

[15] T. Wang, P. Wang, W. Zang, X. Li, D. Chen, Z. Kou, S. Mu, J. Wang, *Adv. Funct. Mater.* **2021**, *32*, 2107382.

[16] K. Srinivas, Y. Chen, B. Wang, B. Yu, X. Wang, Y. Hu, Y. Lu, W. Li, W. Zhang, D. Yang, *ACS Appl. Mater. Interfaces* **2020**, *12*, 31552-31563.

[17] A. Muthurasu, V. Maruthapandian, H. Y. Kim, *Appl. Catal. B: Environ* **2019**, *248*, 202-210.

[18] Z. Liang, W. Zhou, S. Gao, R. Zhao, H. Zhang, Y. Tang, J. Cheng, T. Qiu, B. Zhu, C. Qu, W. Guo, Q. Wang, R. Zou, *Small* **2020**, *16*, 1905075.

[19] Y. Li, W. Wang, B. Huang, Z. Mao, R. Wang, B. He, Y. Gong, H. Wang, *J. Energy Chem.* **2021**, *57*, 99-108.

[20] N. Ma, G. Chen, Y. Zhu, H. Sun, J. Dai, H. Chu, R. Ran, W. Zhou, R. Cai, Z. Shao, *Small* **2020**, *16*, 2002089.

[21] M. Zhou, Q. Weng, X. Zhang, X. Wang, Y. Xue, X. Zeng, Y. Bando, D. Golberg, *J. Mater. Chem. A* **2017**, *5*, 4335-4342.

[22] M. J. Wang, X. Zheng, L. Song, X. Feng, Q. Liao, J. Li, L. Li, Z. Wei, *J. Mater. Chem. A* **2020**, *8*, 14145-14151.
